# Expert perspectives on exposure-response functions for urban health policy: Lessons from a UBDPolicy workshop

**DOI:** 10.1016/j.envres.2025.123150

**Published:** 2025-10-21

**Authors:** Harry Williams, Zorana Jovanovic Andersen, Hanna Boogaard, Søren Brage, Matthew H.E.M. Browning, Samuel Cai, Xuan Chen, Priyanka deSouza, Angel M. Dzhambov, Benjamin Fenech, Gillian Flower, Francesco Forastiere, Leandro Garcia, Antonio Gasparrini, Ulrike Gehring, Alison M. Gowers, Gerard Hoek, Sasha Khomenko, Chris C. Lim, Chenxi Lu, Christina Mitsakou, Andrea Pozzer, Tara Ramani, Charlotte Roscoe, Joseph V. Spadaro, Lambed Tatah, Danielle Vienneau, James Woodcock, Ray Yeager, Belen Zapata-Diomedi, Mark Nieuwenhuijsen, Haneen Khreis

**Affiliations:** a MRC Epidemiology Unit, University of Cambridge, Cambridge, United Kingdom; b Department of Public Health, University of Copenhagen, Copenhagen, Denmark; c Health Effects Institute, Boston, MA, 02110-1940, USA; d Department of Parks, Recreation and Tourism Management, Clemson University, Clemson, SC, USA; e Centre for Environmental Health and Sustainability, Department of Population Health Sciences, University of Leicester, Leicester, United Kingdom; f NIHR Leicester Biomedical Research Centre, University of Leicester, Leicester, United Kingdom; g Institute for Risk Assessment Sciences (IRAS), Utrecht University, Utrecht, the Netherlands; h Department of Urban and Regional Planning, University of Colorado Denver, CO, 80202, USA; i Environmental Health Division, Research Institute at Medical University of Plovdiv, Medical University of Plovdiv, Plovdiv, Bulgaria; j Health and Quality of Life in a Green and Sustainable Environment Research Group, Strategic Research and Innovation Program for the Development of MU - Plovdiv, Medical University of Plovdiv, Bulgaria; k Noise and Public Health, UK Health Security Agency, United Kingdom; l Environment & Health Modelling (EHM) Lab, Department of Public Health Environments and Society, London School of Hygiene & Tropical Medicine, London, United Kingdom; m Department of Health and Social Care, Quarry House, Leeds, LS2 7UE, United Kingdom; n Environmental Research Group, Imperial College, London, United Kingdom; o Centre for Public Health, Queen’s University Belfast, Belfast, Northern Ireland, United Kingdom; p Air Quality and Public Health, UK Health Security Agency, United Kingdom; q Institute for Global Health (ISGlobal), Barcelona, Spain; r Department of Experimental and Health Sciences, Universitat Pompeu Fabra (UPF), Barcelona, Spain; s CIBER Epidemiología y Salud Pública (CIBERESP), Madrid, Spain; t Zuckerman College of Public Health, The University of Arizona, Tucson, AZ, USA; u Potsdam Institute for Climate Impact Research (PIK), Potsdam, Germany; v Sustainability Economics of Human Settlements, Technical University Berlin, Berlin, Germany; w Max Planck Institute for Chemistry, Mainz, Germany; x The Cyprus Institute, Nicosia, Cyprus; y Texas A&M Transportation Institute, Texas A&M University System, TX, USA; z Environmental Systems and Human Health, Oregon Health & Science University–Portland State University School of Public Health, Portland, OR, USA; aa Division of Oncological Sciences, OHSU Knight Cancer Institute, Oregon Health & Science University, Portland, OR, USA; ab Spadaro Environmental Research Consultants (SERC), Philadelphia, PA, USA; ac WHO Consultant (European Centre for Environment and Health, Bonn, Germany); ad Swiss Tropical and Public Health Institute, Allschwil, Switzerland; ae University of Basel, Basel, Switzerland; af University of Louisville, School of Medicine, Division of Environmental Medicine, USA

**Keywords:** Exposure-response functions, Health impact assessment, Air pollution, Transport noise, Greenspace, Non-optimal temperature, Physical activity

## Abstract

Policy-makers require robust, quantitative evidence in order to better align urban and transport planning practices with public health goals. Epidemiologically derived exposure-response functions can quantify the association between urban health determinants and human health outcomes. They are therefore a crucial input in quantitative health risk assessments, providing to policy-makers actionable evidence on how healthier, more sustainable cities may be achieved.

The Urban Burden of Disease Policy (UBDPolicy) project convened a two-day workshop to discuss recent developments, ongoing challenges, and future directions for exposure-response functions and their application to quantitative health risk assessment. The workshop discussions centred around air pollution, transport noise, non-optimal temperature, greenspace and physical activity as primary pathways through which urban and transport planning impact human health. Based on this workshop, we provide an expert-guided perspective on how to enhance both our conceptual understanding of exposure-response functions and their practical application in urban health risk assessment. We also identify pathway-specific as well as cross-cutting (e.g., quantifying multiple exposures, need for population sub-group evidence) research needs relevant to environmental health more broadly. We propose several future research directions as an agenda for advancing urban environmental health.

## 1. Introduction

Suboptimal urban and transport planning can result in increased exposure to air pollution, noise, and excess heat, resulting in large but preventable mortality and morbidity burdens (Khomenko et al., 2021, 2022; Iungman et al., 2023). Conversely, by increasing access to greenspace and levels of physical activity (e.g., by expanding cycling and pedestrian networks), a large number of premature deaths could be prevented annually (Barboza et al., 2021; Mueller et al., 2018). Better urban and transport planning practices can improve public health (Nieuwenhuijsen, 2021), however, in order to take effective action, decision-makers require robust evidence aligning health needs and outcomes with social, environmental, economic and commercial determinants (WHO, 2023).

Quantitative evidence on the population health impact of environmental factors, exposures, policies or programmes can be produced through a quantitative health risk assessment, such as a burden of disease study or a health impact assessment (HIA) (Rigaud et al., 2024). In Europe, quantitative HIAs have previously demonstrated 8–20% of annual mortality burdens to be associated with urban and transport planning related determinants (Khomenko et al., 2020; Mueller et al., 2017). To conduct a quantitative HIA, an external factor must first be deemed causally or likely to influence human health, and an exposure-response function (ERF), representing the quantitative association between an exposure and health outcome, must be available (Rigaud et al., 2024). The choice of the ERF is a key step in the HIA process (Mueller et al., 2023) and an important factor influencing the range of outcomes, meaning it is critical to select the most appropriate ERF for its intended purpose.

In September 2024, UBDPolicy (https://ubdpolicy.eu/) convened an international and multidisciplinary group of experts and stakeholders to explore recent advancements, current challenges, remaining gaps, and future directions for ERF research, and their implications for urban HIA. This manuscript details the content and discussions of this workshop into a comprehensive research agenda that aims to re-orient researchers toward enhancing both our conceptual understanding of ERFs, and their effective and practical application in HIAs. Through doing so, we aim to advance the confidence in quantitative evidence that supports more effective urban and transport planning practices to protect and improve public health. Previous reviews (Woodcock et al., 2025; Rigaud et al., 2024; Forastiere et al., 2024) have provided discussion on ERFs, but only within the broader context of quantitative health risk assessment where they are discussed alongside other relevant inputs and modelling assumptions and choices. We instead offer an in-depth focus on the estimation and application of ERFs given their significance in environmental health research, practice, and policy, providing actionable recommendations for researchers to apply in ongoing and future work.

**Fig. 1.**
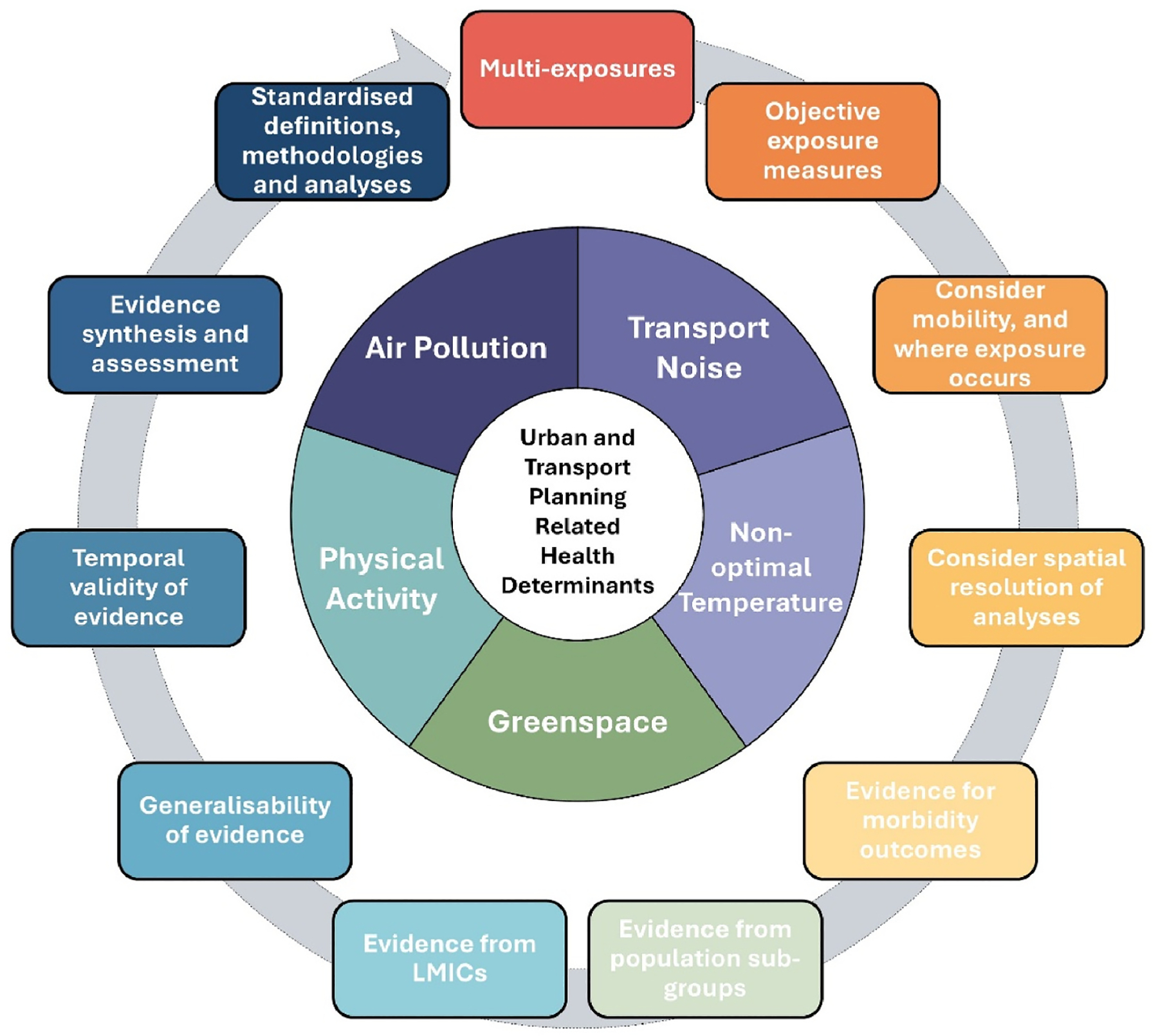
Overview of urban and transport planning related health pathways included in commentary, along with cross-cutting research needs identified.

The discussions were structured as follows. First, we identified pathway-specific research needs, focussing on ambient air pollution, transport noise, non-optimal temperature, lack of greenspace and physical activity as key pathways through which suboptimal urban and transport planning may influence public health and where the evidence now allows for quantification (e.g., Glazener et al., 2021). Second, we identified several cross-cutting research needs (Fig. 1) applicable to all pathways and environmental health research more broadly. Finally, we considered how ERFs inform the HIA process, and implications for policy processes. We use the term ERF throughout due to its widespread applicability and use. However, when relevant, we also refer to other related terms (Table 1). Key themes were derived from expert-led discussions before and during the workshop and iteratively refined while drafting the manuscript. While no formal consensus method was applied, thematic agreement was reached by prioritising recurrent points of discussion and peer-review comments, with broad multidisciplinary agreement and refinement across 30+ co-authors and multiple revision rounds.

Importantly, we acknowledge this work as an unexhaustive, non-systematic account of a large and expanding evidence base. However, by leveraging participants’ subject-matter expertise to build upon workshop discussions, we highlight several prominent knowledge gaps and research needs (Table 2). This work additionally serves to encourage holistic consideration of a diverse body of evidence often discussed in isolation, to the detriment of integrated evidence assessment and application.

## 2. Discussion

### 2.1. Pathway-specific research needs

#### 2.1.1. Air pollution

Ambient air pollution remains a primary environmental contributor to mortality and morbidity worldwide (Kasdagli et al., 2024; Orellano et al., 2024; Forastiere et al., 2024a) with broad consensus based on decades of scientific literature confirming air pollution as a major global public health risk factor (Brauer et al., 2024; Boogaard et al., 2019). Most workshop discussions centred around fine particulate matter (PM_2.5_) due to its public health significance, however we recognise that other ambient air pollutants including black carbon (BC), ozone (O_3_) and nitrogen dioxide (NO_2_) also contribute to the global disease burden (Strak et al., 2021; Brauer et al., 2024). Based on substantive epidemiological evidence for major health outcomes, well-established ERFs are available, however, several important and unresolved questions remain (Boogaard et al., 2024; Forastiere et al., 2024b) that we discuss here.

##### 2.1.1.1. Evolving risks (stronger recent effects for PM_2.5_).

Recently published meta-analyses (Orellano et al., 2024) show moderately stronger associations between PM_2.5_ and all-cause or non-accidental mortality, indicating that the already large current burdens of air pollution might be underestimated when using previous ERFs (Chen and Hoek, 2020; Hoek et al., 2013). The epidemiological evidence published between the systematic reviews by Hoek et al. (2013) and Orellano et al. (2024) results in an estimated relative risk (RR) for all-cause mortality per 10 μg/m^3^ increase in PM_2.5_ exposure that is 50 % higher (relatively) than the previous estimate (RR = 1.062 vs. 1.095, respectively). Orellano et al. (2024) also found higher PM_2.5_ summary estimates for all-cause mortality than those reported by Chen and Hoek (2020) (RR = 1.095 vs. 1.08), albeit with wider 95 % confidence intervals due to the large increase in the number of recent studies (60 additional studies). This difference may be explained by several possible reasons. Improved resolution of air quality data might have allowed for better capturing of exposure contrasts, and likely less exposure measurement error. It is often assumed that exposure measurement error would bias the associations towards the null, albeit exposure measurement error may be complex (Samoli and Butland, 2017; Sheppard et al., 2012). It may also be explained by other factors such as changes in PM_2.5_ composition over time, in the pollutant mixture correlated with PM_2.5_ concentrations and/or by more recent studies in settings such as Europe and North America where air pollution levels are relatively low, which paired with a supra-linear ERF curve results in a larger relative effect per additional exposure unit at low compared to higher concentrations (Boogaard et al., 2024).

In contrast, the risk estimate for NO_2_ has not exhibited an increasing trend, with estimates not changing between recent comparable systematic reviews and meta-analyses conducted in 2013 (Hoek et al., 2013) and 2024 (Kasdagli et al., 2024) (RR = 1.05 and 1.045 per 10 μg/m^3^, respectively), and a notably smaller RR in a 2020 review (RR = 1.02 per 10 μg/m^3^) (Huangfu and Atkinson, 2020). This difference in trend warrants further examination to help clarify the factors that may affect associations between specific air pollutants and health outcomes, and the extent of this influence.

##### 2.1.1.2. Unknown heterogeneity (shape of the PM_2.5_ exposure-response curve).

**Fig. 2.**
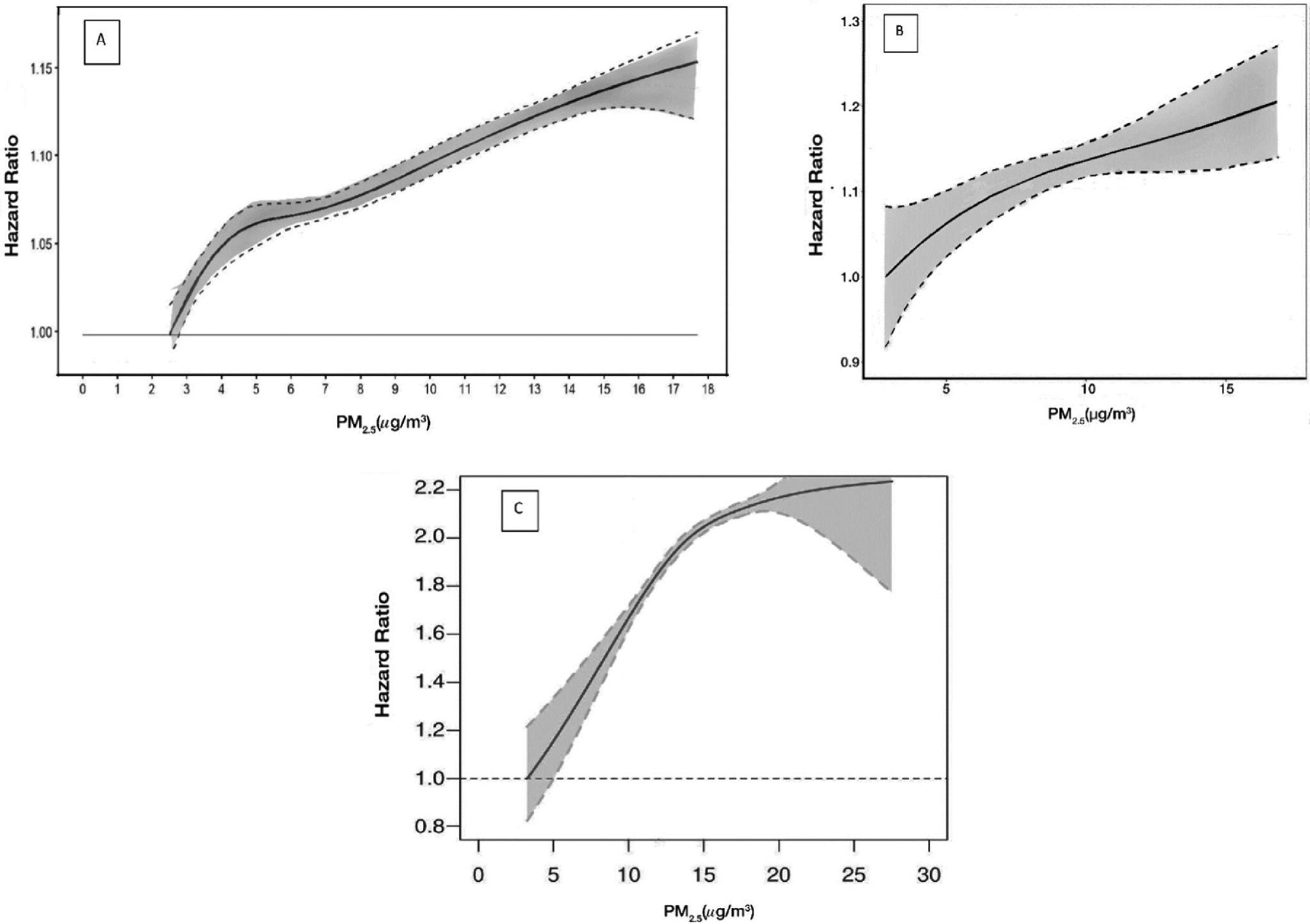
CRFs for associations between long-term exposure to PM_2.5_ and all-cause or nonaccidental mortality in (A) the Canadian MAPLE study; (B) U.S. Medicare study; and (C) European ELAPSE Pooled cohort. Shaded area corresponds to the 95 % CI. From Boogaard et al. (2024).

While there is consensus that current epidemiological studies have not provided evidence of a threshold concentration below which no effect occurs (Orellano et al., 2024), recent research has highlighted substantial heterogeneity in the shape of the CRF for PM_2.5_ at low levels (Fig. 2), with different shapes observed in different regional cohorts (near linear in the U.S., and supra-linear in Europe and Canada) (Boogaard et al., 2024). Somewhat surprisingly, the variability in the magnitude and shape of the association across the Canadian, U.S. and European studies was reduced only slightly in a harmonized analysis (Chen et al., 2023a). Similarly, there is some evidence of heterogeneity in the shape within regions (e.g., between different European cohorts) (Brunekreef et al., 2021).

It is not yet clear to what extent these results may be due to sampling variability, differences in populations and their responses, the toxicity of the air pollution mixture, and/or the statistical methods used, and therefore warrant further examination, particularly when considering the significant implications that a supra-linear CRF could have for quantifying health effects at low-levels of exposure (Weichenthal et al., 2022). For now however (as suggested by the UK Committee on the Medical Effects of Air Pollutants (COMEAP)), the current evidence remains insufficient to recommend any change from the current assumption of a linear CRF relationship when quantifying the impacts associated with long-term exposure to PM_2.5_, although there is value, in research settings, to investigate the influence of using different CRF shapes, as sensitivity analyses (COMEAP 2025 (in preparation)).

##### 2.1.1.3. Drivers of heterogeneity (PM_2.5_ components).

PM_2.5_ is heterogenous, varying by source, size and chemical composition across regions (McDuffie et al., 2021; van Donkelaar et al. 2019). Research suggests that a wide range of PM_2.5_ components are associated with adverse health effects (Hao et al., 2023; COMEAP, 2022b), and varying exposure-response curves (Chen et al., 2024a). Despite this heterogeneity, epidemiological studies have found consistent associations with mortality and morbidity in very different settings globally (Chen and Hoek, 2020), with such evidence underpinning the World Health Organization’s (WHO’s) 2021 revised guidelines for PM_2.5_ (WHO, 2021). Relatedly, the consistency of adverse effects observed across diverse settings provides support for the usefulness of PM_2.5_ mass concentrations as a metric for ambient particles, which remains useful for quantitative HIAs (COMEAP, 2022a). As concluded by the United States Environmental Protection Agency (U.S. EPA) in their 2019 Integrated Science Assessment (ISA) for particulate matter, there is currently insufficient evidence for different PM components being more closely related to health outcomes than PM_2.5_ mass and no individual PM_2.5_ component or source is a better predictor of mortality than PM_2.5_ mass (US EPA, 2019), although this evidence needs updated synthesis and assessment. Future studies should explore how PM_2.5_ sources and composition are evolving in response to anthropogenic changes, particularly to transport decarbonisation and electrification, climate change, and wildfires (Xu et al., 2023; COMEAP, 2020). Source apportionment studies may help further elucidate sources of PM and its components, with practical application for epidemiology and HIA (Shan et al., 2024).

Similarly, PM_2.5_ occurs within a mixture of other pollutants such as volatile organic compounds (VOCs), and O_3_ and NO_2_ gases, that not only interact with and impact PM_2.5_ toxicity (e.g., Weichenthal et al., 2017), but are themselves associated with well-established independent health effects. Owing to substantial evidence delineating these independent health effects, WHO updated recommendations on air quality guideline levels for gaseous pollutants, primarily NO_2_ and O_3_ in 2021. These gaseous pollutants also exhibit substantial global variation (McDuffie et al., 2020), and these differing correlations may partly explain observed heterogeneities in the magnitude and shape of reported single pollutant PM_2.5_ ERFs. These complexities benefit from more advanced approaches (e.g., the pollutant mixture complexity index) to better separate risks of PM_2.5_ from risks of gaseous pollutants on mortality (Masselot et al., 2024) or delineate additive, synergistic or antagonistic effects which remain largely unknown. The development of multi-pollutant statistical approaches (e.g., Chen et al. (2024b) discussed in more detail later) remains an active area of research. While numerous advanced approaches have been developed, particularly for omics analyses and in studies of the exposome (Stafoggia et al., 2017; Agier et al., 2016; Chadeau-Hyam et al., 2013), further progress remains necessary, especially in the analysis of complex pollutant mixtures, and in the integration of epidemiological and toxicological data to better interpret intricate exposure patterns and their health effects (Savitz and Hattersley, 2023).

#### 2.1.2. Noise

Transport noise - primarily from road traffic, railway and aircraft - is a significant environmental risk factor for which the epidemiological evidence of adverse health effects continues to grow. As highlighted in both a recent European Environment Agency (EEA) report (EEA, 2025; Engelmann et al., 2023) and systematic review (Pershagen et al., 2025), consistent associations are now observed for long term health effects including all-cause mortality, incidence of cardiovascular diseases and diabetes. Adverse effects start below current WHO Environmental noise guidelines for Europe (WHO, 2018), and emerging evidence indicates road and railway noise may increase risks for dementia, breast cancer and tinnitus (EEA, 2025; Sørensen et al., 2023).

##### 2.1.2.1. Assessment methods (noise exposure-estimation).

The current state of the art for noise exposure assessment are source-specific emissions plus propagation methods that estimate exposure at façade points on buildings or a fine lattice. These models consider noise emissions from different vehicle types, volumes and speeds, together with sound absorption and reflections from ground terrain, buildings and other structures such as noise barriers - see for example the Nord2000 method (Kragh et al., 2023). Within agglomerations of more than 100,000 persons and near major roads, railways and airports, strategic noise mapping by transportation source is mandatory for European countries under the Environmental Noise Directive (END). However, historically, strategic noise maps developed under END differ substantially across countries, due to different exposure assessment methods and variations in the resolution of input data, resulting in many being of low quality (Khomenko et al., 2022). Similarly, few noise maps employ continuous noise exposure ranges, more frequently categorising by 5-dB noise bands that may misclassify exposure levels and increase uncertainty (Khomenko et al., 2022). Burden of disease estimates based on END strategic noise maps, that only consider agglomerations and major noise sources (e.g., major roads, railways and airports) and do not include lower levels of noise exposure, may be underestimated (EEA, 2025; Aasvang et al., 2023; Jephcote et al., 2023).

Having modelled noise exposure at the façade on the floor of residence vs. using a spatial grid for residential exposure assessment can also substantially influence health estimates (Vienneau et al., 2019). While the preferred façade exposure estimates have been utilised in settings such as Denmark and Switzerland to produce high resolution datasets, such spatially resolved models are not widely available and remain underutilised more widely (Vienneau and Wunderli 2023). More broadly there remains a paucity of exposure data outside Europe (The Lancet Regional Health - Europe 2023), though geospatial approaches (e.g., land-use regression) using monitoring data may be suited to scale up noise exposure assessment in epidemiological studies (Roscoe et al., 2023), particularly for low- and middle-income countries (Chen et al., 2023b).

##### 2.1.2.2. Expanding classifications (source-specific transport noise).

In a typical urban environment, people are exposed to multiple noise sources (e.g., transport, construction, commercial, entertainment). Transport noise, for which most evidence is available, is typically further evaluated by source, for which the most compelling evidence comes from road traffic, at least partly related to the number of studies (Engelmann et al., 2023). In lieu of source-specific noise ERFs for all outcomes, which are necessary to reflect the different acoustic characteristics of sources (Wunderli et al., 2016), discussions on the appropriateness of applying pooled estimates representing total transport noise remain ongoing. Based on the assumption that the biological mechanisms involved may be similar for different noise sources (Sørensen et al., 2023), in some circumstances estimates pooling different noise sources may be appropriate. For example, a recent EEA report proposes the use of a pooled effect estimate for transport noise to estimate cardiovascular risks of rail and aircraft noise for which the evidence is inadequate (Engelmann et al., 2023). This however is not appropriate for all sources nor all outcomes (e.g. annoyance, for which source-specific curves are available).

While the effects of environmental (including transportation) and occupational noise exposure are relatively well researched, some noise sources are better studied than others (SheikhMozafari et al., 2025), and new sources continue to emerge (Willis et al., 2024). More is known, for example, about the broad range of effects of road traffic noise, that affects nearly everyone, compared to railway noise. Compared to, for example, the more advanced work on source apportionment that has been undergone for air pollution, more effort is needed to identify sound sources if using measurements instead of engineering models as a basis for exposure assessment. This may be of particular policy relevance given, for example, the potentially beneficial effects of pleasant and calm soundscapes for health and wellbeing (Kong and Han, 2024). Additionally, there is a need to recognise that the sources and characteristics of transportation noise likely differ by study setting and, therefore, the current evidence base that is largely derived from European studies may not be generalisable to other study settings with differing noise profiles. For example, in low- and middle-income country cities daytime and night-time noise levels can frequently exceed those in European cities (Chen et al., 2023c), and additional evidence from sub-Saharan Africa based on measurements highlights heterogeneous noise sources and unique soundscapes (Clark et al., 2021; Osei and Effah, 2022). Further research is also needed to better understand how other contextual factors (e.g., built environments, population densities, underlying population and health characteristics) may influence noise characteristics and their effects.

In recognition that individuals can be exposed to multiple sources of transportation noise (e.g., road, railway, aircraft) simultaneously, there is also a need for studies to investigate the health effects of combined noise exposure for which less is known, and to determine which models are best suited to predict combined risks (Sørensen et al., 2023; Seidler et al., 2019).

##### 2.1.2.3. Confounding and effect modification (noise and air pollution).

Given that transport represents a major source of both noise and air pollution, research on transport noise published in the last decades almost always considers air pollution at least as a potential confounder. Very few studies have looked at interactions (Eminson et al., 2023). Generally, studies have found that associations between noise and health outcomes are independent of air pollution (Eminson et al., 2023). For example, in the Swiss National Cohort, Héritier et al. (2019) find transport noise to be independently associated with myocardial infarction mortality, even after adjustment for NO_2_ and/or PM_2.5_. This is not observed in all contexts (e.g., in analysis of the UK Biobank, Hao et al. (2022) consistently find noise effect estimates reducing after adjustment for PM_2.5_). When considering the reverse, the evidence is mixed, with some studies observing associations with traffic-related air pollution to be attenuated after adjustment for noise, and some observing no change in air pollution effect estimates (Rugel and Brauer, 2020). Many studies on air pollution however do not adjust for confounding by noise at all. Future research should explore the possible occurrence of effect transfer, which can arise when exposure is less accurately estimated for one exposure compared to another (Evangelopoulos et al., 2021). This may be particularly relevant for noise, considering that advanced sound propagation models are available in only a limited number of settings.

#### 2.1.3. Greenspace

Current available evidence for the extent of local greenspace indicates protective effects for a growing number of long-term health outcomes (e.g., all-cause and stroke mortality, CVD morbidity, mental health), but remains limited or inconclusive for many others (Sakhvidi et al., 2023; Yang et al., 2021; Rojas-Rueda et al., 2019). Generally, greenspace ERFs exhibit heterogeneity (varying substantially by type of greenspace, outcome examined, and contexts) and are consequently a significant source of uncertainty in greenspace HIA (Barboza et al., 2021).

##### 2.1.3.1. Defining and categorising greenspace.

Greenspace, which itself exhibits significant variability, lacks a common unifying definition, with definitions often changing between disciplines (Taylor and Hochuli, 2017). The definition of greenspace used can alter observed outcomes (e.g., Klompmaker et al., 2018). Furthermore, greenspace can be represented by a variety of different metrics and indicators, all measuring different aspects of the environment (e.g., greenness, vegetation type and cover, access to and use of open spaces) (Liu et al., 2023; Vilcins et al., 2024a,b), and the way in which greenspace is measured and classified can impact statistical associations (Mitchell et al., 2011). The substantial variation in what is considered greenspace and qualities therein (Kondo et al., 2018) have important implications for HIA, as the definition of greenspace used to derive a particular ERF may not necessarily match definitions used in policies and HIA. While definitional decisions will depend on the research question and intended application, researchers should still aim to provide a meaningful definition that both qualifies and quantifies how greenspace is defined (e.g., examples presented by Taylor and Hochuli, 2017), for which construct-based approaches may be additionally useful (Lee et al., 2025). For future research, it is feasible to obtain and evaluate typologically-distinct and context-specific metrics of greenspaces across many urban areas (e.g., canopy and grasses within or proximate to residential, near-road, and park contexts) (Browning et al., 2024).

To date, HIAs have primarily relied on normalised difference vegetation index (NDVI) as a simple and inexpensive metric of vegetation extent despite its many limitations such as the inability to accurately characterise how individuals access, interact with and experience greenspace (Donovan et al., 2022; Holland et al., 2021). For some outcomes, contact with and use of greenspace may be more important predictors of health than NDVI or proximity-based metrics (Holland et al., 2021; Kruize et al., 2020; White et al., 2021), therefore more ERFs should be derived using metrics that more accurately reflect them (e.g., White et al., 2019). Importantly, NDVI also lacks specificity to differentiate fundamental aspects of green cover such as trees, grasses, quality, and visibility, and does not differentiate between publicly accessible and private greenspace. Such aspects can influence observed health effects, and are therefore necessary to consider to enable targeted health intervention (Yi et al., 2025; Odebeatu et al., 2024). Future research needs more sophisticated and precise exposure metrics. This is partially due to a need to consider which metrics may more accurately reflect specific mechanistic pathways of interest (for example when addressing noise attenuation, biomass metric may be more appropriate than NDVI). This will further require greater consideration of characteristics of greenspace morphology, including quality, size, shape, fragmentation, connectedness, aggregation, and diversity (Wang et al., 2024). Spatial resolution, which can differ substantially (e.g., NDVI resolution from MODIS (250 m^2^) and Landsat 8 (30 m^2^)), should also be considered, and decisions made based on context-relevant pathways (Jimenez et al., 2022).

There is a further need to consider potential differences between objectively measured and perceived metrics of exposure, which do not always align (Marselle et al., 2021; Leslie et al., 2010). Some evidence indicates perception may matter more for certain pathways (e.g., Dzhambov et al. (2018) found associations of mental health to vary depending on whether subjective or objective measurements of greenspace were used). Alternative metrics that more accurately capture how greenspace is perceived by individuals may therefore be better suited (e.g., Viewshed Greenness Visibility Index (VGVI) (Labib et al., 2021), when metrics relevant to harm mitigation are not being considered. Perceptions of greenspace quality (e.g., attractiveness, cleanliness, and safety) may also impact health outcomes (Knobel et al., 2021) with differing patterns of associations depending on outcome (Nguyen et al., 2021). Yet, perceptions of quality may also vary based on individual and community-level culture and values. Evidence is lacking for other qualities, particularly the identification of needs-specific or culturally appropriate amenities, and there is a need for more longitudinal and experimental studies (Nguyen et al., 2021).

##### 2.1.3.2. Drivers of heterogeneity (pathway- and context-specific greenspace).

**Fig. 3.**
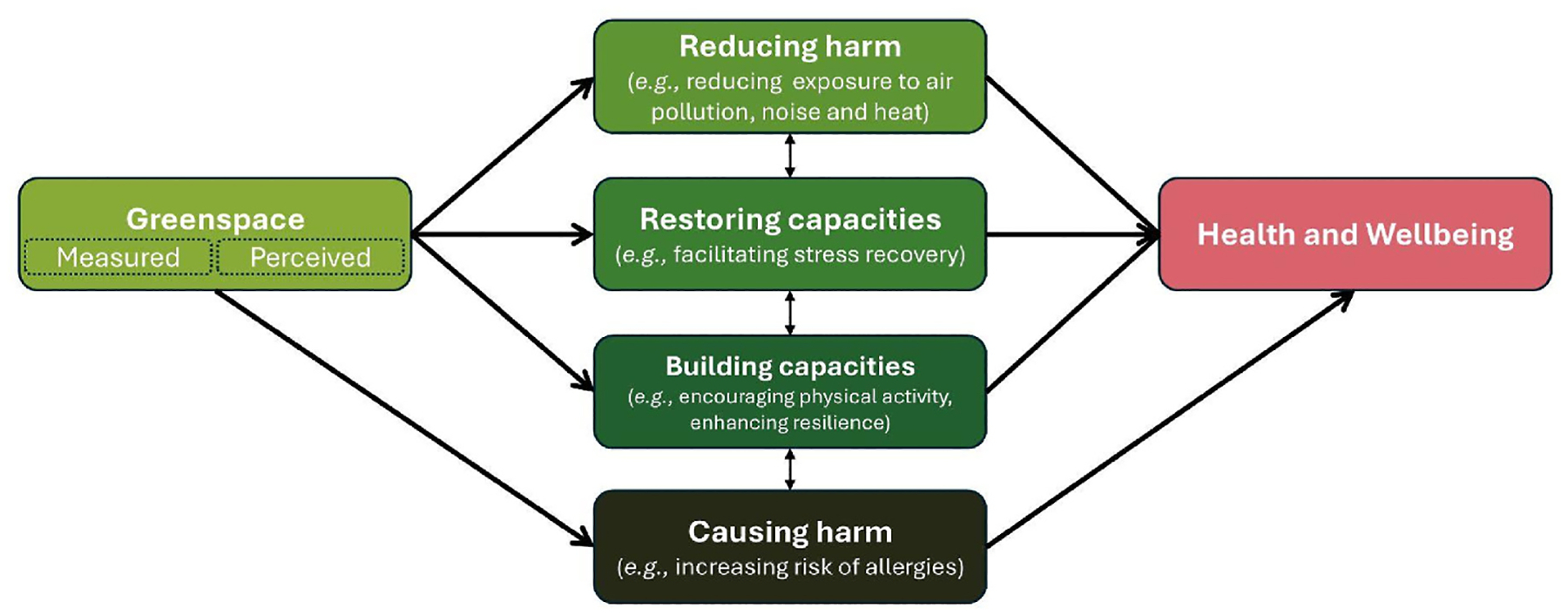
Pathways linking greenspace to human health. Adapted from Marselle et al. (2021) and Markevych et al. (2017).

Greenspace, and nature more broadly, impacts health and wellbeing through four mechanistic pathway domains (each comprised of many individual pathways) (Fig. 3). These mechanistic pathways may be important drivers behind varying greenspace-health associations that further differ by context. For example, in an urban environment the effects of greenspace may be more driven through mitigation (e.g., reducing harm from heat, air pollution, noise) than in rural contexts (Browning et al., 2022). HIAs should therefore attempt to separate out different fundamental contexts (e.g., high burden urban environments, low burden urban environments, rural). Further research may also improve our understanding of how ERFs differ between environmental contexts and how they may further differ among sub-groups of the population. These different pathways and contexts are further complicated by the numerous concurrent mediators and moderators operating at all social and spatial levels (Marselle et al., 2021; Dzhambov et al., 2020) that are increasingly complex to model and incorporate within HIA. For example, sex and gender may modify the association between greenspace and health (Sillman et al., 2022) however more robust empirical studies are required to grow the evidence base for this and many other moderators/mediators for which less is known (e.g., modifications by personal traits, or mediation by physical activity) (Sakhvidi et al., 2023). There are many seemingly important moderators/mediators for which there is yet no consensus on whether they are essential to measure, such as nature-relatedness (i.e., people’s connection to nature) (Dean et al., 2018), or nature contact during commuting to (Zijlema et al., 2018) or during work (Lygum et al., 2023). Research on greenspace, and nature more broadly, continues to evolve, incorporating new evidence (e.g., the recently proposed biopsychosocial resilience framework (White et al., 2023)) as it emerges. Overall, greenspace research more routinely integrates pathway analyses to statistically disentangle the role of different mediators, which should also be replicated for other environmental exposures where it received less attention (Dzhambov et al., 2020).

##### 2.1.3.3. Expanding classifications (nature is not always ‘green’).

Most available evidence for nature focuses on greenspaces, and increasingly blue space (White et al., 2020). However, there is a need to move beyond typical definitions of ‘green space’ and ‘blue space’ in recognition that nature is diverse, with emerging research demonstrating other landscapes such as ‘white space’ (snow-covered) or ‘brown space’ (rock-covered or deserts) may pose health benefits or risks not currently captured (Li et al., 2023). Further, each of these space categories facilitates exposure to other types of nature such as natural soundscapes, microbial colonisation, biogenic chemicals, animal interactions, and many other elements of nature contact. The biodiversity of natural landscapes, which can influence human health but is not as extensively researched (Marselle et al., 2021), requires greater consideration. Individual perceptions of these specific landscapes and contacts may influence health benefits (e.g., deserts can activate restorative pathways among desert residents, but others may view these landscapes as harsh/barren and therefore not receive the same benefit) (Yin et al., 2022). The ‘naturalness’ of greenspace and other landscapes also requires further consideration, with research demonstrating its importance for improved mental wellbeing (Bressane et al., 2024). Similarly, however, naturalness may be perceived differently by individuals (Hoyle et al., 2019; Hwang and Roscoe, 2017). Seasonality, which can significantly influence the physiognomy of green and other natural environments, is another commonly overlooked dimension (Bell et al., 2017) that can influence health and physical benefits (Zhou et al., 2022). Therefore, the temporality of chosen exposure metric (e.g., summertime maximum or annual average) may influence results, requiring careful consideration on which to select based on the pathways of relevance.

#### 2.1.4. Non-optimal temperature

Non-optimal temperatures (both heat and cold) exert substantial mortality impacts worldwide, though most of the current mortality burden can be attributed to cold (Masselot et al., 2023; Zhao et al., 2021). Due to climate change, heat-related health burdens are expected to increase, and prevailing regional disparities in these burdens will continue to widen (García-León et al., 2024). Differential patterns of risk are also observed according to age, with risk increasing with age, and by cause (e.g., cardiorespiratory causes show stronger effects than non-cardiorespiratory causes) (Scovronick et al., 2024). Health estimates for temperature-related risk vary across cities due to the numerous climatic, environmental, and socio-economic factors which influence vulnerability to heat and cold (Sera et al., 2019). Thus, while the evidence for short-term effects of heat and cold is robust, the use of a meta-analytic summary estimate, as for air pollution, noise and greenspace, is not supported. City or region-specific ERFs should be used in HIA.

##### 2.1.4.1. Assessment methods (modelling complex temperature-related effects).

The temperature-mortality relationship comprises the effects of exposure to heat and cold, both of which contribute to excess mortality. The minimum mortality temperature (MMT) is often used to represent the ‘optimum’ temperature at which the risk of mortality is lowest. It reflects adaptability to local climate, and differs widely across cities (Tobías et al., 2021). Modelling this non-linear (typically J- or U-shaped) temperature-mortality association is complex, characterised by different lag periods, that further vary substantially between populations due to differences in acclimatisation, susceptibility, age structure, access to resources and local public policies (Guo et al., 2014). These complexities require sophisticated statistical approaches and large amounts of historical data (Gasparrini et al., 2015). This is exemplified in Masselot et al. (2023) where an advanced three-stage modelling framework was developed and used to provide comprehensive risk estimates and mortality impact assessment of non-optimal temperature across 854 European urban areas. Their work also offers clear directions for future developments, including Bayesian applications and machine learning to improve risk spatialisation and predictive performances, and the adoption and application of their framework to other environmental stressors.

##### 2.1.4.2. Evolving risks (population adaptation to and long-term effects of non-optimal temperature exposure).

The health effects of short-term exposure (days to weeks) to non-optimal temperatures are well established, with robust estimates routinely employed in HIA (Iungman et al., 2025; Khomenko et al., 2025). Conversely, there are very few studies investigating associations between long-term temperature exposure and health (Zafeiratou et al., 2021), and less physiological evidence on what the effects of long-term exposure to non-optimal temperature could be. Establishing the presence of an independent effect of long-term exposure is methodologically challenging due, in large, to population adaptation processes which occur over time (Zafeiratou et al., 2021). These processes, which may be due to behavioural, physiological, or societal adjustments, may lead to the mitigation of long-term health effects, and are likely to vary by context as demonstrated by substantial global variability in the MMT, an important indicator of adaptability to climate (Tobías et al., 2021). A deeper understanding of these adaptive mechanisms is crucial, especially in the context of climate change and projection of future temperature-related health burdens, for which there is limited methodological guidance (Rai et al., 2022). Further research, utilising innovative designs and longer series is warranted.

The current evidence base for long-term exposure to non-optimal temperatures is sparse, and existing studies exhibit substantial heterogeneity regarding study design, outcome considered, temperature metric and exposure duration. Recent evidence from a European-wide small-area study indicates potentially independent effects of long-term exposure to non-optimal warm periods, beyond those of short-term exposure, although these results were again highly heterogeneous across geographic areas and temperature metrics (Zafeiratou et al., 2025). A multi-country study explored the timescale of heat-mortality associations, suggesting that most of the long-term effects can be explained by the cumulation of short-term inputs (Armstrong et al., 2017). However, this question must be clarified by further longitudinal research, particularly through the application of non-ecological, longitudinal cohort studies that cover a range of geographical areas and incorporate individual-specific information (e.g., age, socioeconomic status, pre-existing conditions) as well as area-specific characteristics.

##### 2.1.4.3. Confounding and effect modification.

There is strong evidence linking temperature-related mortality and effect modification by individual-level factors such as age, sex, and occupation (Son et al., 2019). Research has also demonstrated how environmental factors such as greenspace and air pollution modify temperature-related effects (Song et al., 2024; Wicki et al., 2024; Stafoggia et al., 2023; Choi et al., 2022) however, the evidence for community-level effect modifiers (e.g., population density, healthcare facilities) is limited and requires further research (Son et al., 2019). Evidence on effect modification is also limited for cause-specific outcomes (Zafeiratou et al., 2023), and more research is needed to understand behavioural responses (Folkerts et al., 2022). Examination of biobanks and electronic health records could also reveal further insight into modifiers such as genetic susceptibility and medication usage, respectively.

##### 2.1.4.4. Evolving risks (climate change and climate justice).

Climate change impacts, including unprecedented global warming and extreme events such as heatwaves (IPCC, 2023) and cold spells (Hanna et al., 2024), are likely to worsen worldwide. One third of heat-related deaths have already been attributed to anthropogenic climate change (Vicedo-Cabrera et al., 2021), which is further exacerbating existing heat-related health and economic burdens (García-León et al., 2024; van Daalen et al. 2024). Recent evidence also demonstrates that increases in heat-related deaths consistently exceed any decrease in cold-related deaths, resulting in net temperature-related health burdens increasing, even under scenarios of high mitigation and adaptation (Masselot et al., 2025). Incidentally, the question of population-adaptation remains a substantial source of uncertainty in projections of temperature-related health burdens (Rai et al., 2022). While there are a variety of approaches for quantitatively incorporating adaptation into impact assessments, there remains no best practice (Rai et al., 2022; UK Health Security Agency, 2024). Additionally, methods for including adaptation are often not grounded in empirical evidence (Cordiner et al., 2024), and there remains additional uncertainty relating to the appropriateness of applying current models of adaptation to future impacts that are extending beyond historical experiences (Vanos et al., 2020). The impact of population ageing, a global trend, is also likely to be a crucial driver for future temperature-related risks that will amplify the increasing impacts of extreme heat (Chen et al., 2024c), warranting further research. Beyond the independent physiological effects of heat exposure, long-term changes in heat and other weather patterns may also influence the foundational behavioural and social determinants of health. These include physical activity, transportation, access to facilities and services, greenspace typologies and biodiversity, economic and productivity loss, and social cohesion (Ragavan et al., 2020; Berry et al., 2010; Moser and Hart 2015).

There also remains a need for research into social inequities and environmental injustice of climate on health, moving beyond just physiological characteristics (e.g., age, underlying health conditions). It should consider the underpinning forms of exclusion, oppression and exploitation that are driving unequal health impacts in historically marginalised and minoritised groups who have contributed the least to climate change, are most exposed to its impacts and have the worst access to protective interventions (Kotsila and Anguelovski 2023). Researchers should more closely examine ongoing and intersecting oppressive structures that underpin existing health inequities but also interact with climate change to exacerbate them (Deivanayagam et al., 2023).

#### 2.1.5. Physical activity

Physical activity can be considered a product of built environment and policy decisions, representing a pathway through which human health is influenced by urban and transport planning, as conceptualised through several well-established frameworks (Glazener et al., 2021; Tonne et al., 2021; Nieuwenhuijsen, 2020). For example, compact, mixed-land use design and active transport infrastructure can promote physical activity levels and improve public health (Cerin et al., 2022; Khreis et al., 2024; Kärmeniemi et al., 2018). Similarly, urban interventions such as low-traffic neighbourhoods or 15-min cities aim to simultaneously reduce car usage and improve urban environments by promoting physical activity, enacting multiple co-benefits (Nieuwenhuijsen et al., 2024; Xiao et al., 2024). Participating in regular physical activity results in health benefits across all ages and abilities. Recent systematic reviews have shown non-linear dose-dependent associations between higher physical activity volumes and decreased risk of a wide range of mortality, cardiovascular disease, cancer (Garcia et al., 2023) and mental health outcomes (Pearce et al., 2022).

##### 2.1.5.1. Defining and categorising physical activity.

Physical activity is a diverse behaviour, comprising several activity types and domains, varying by intensity, duration and frequency, resulting in heterogeneity in operational definitions across studies (Piggin, 2020). There are also multiple ways to measure and report physical activity, and different decisions and approaches to estimate DRFs. Consequently, many physical activity studies are challenging to compare, indicating a need for comprehensive data harmonisation strategies (Garcia et al., 2023; Pearce et al., 2022; Gates et al., 2017) and standardised methodological decisions and approaches across outcomes.

**Fig. 4.**
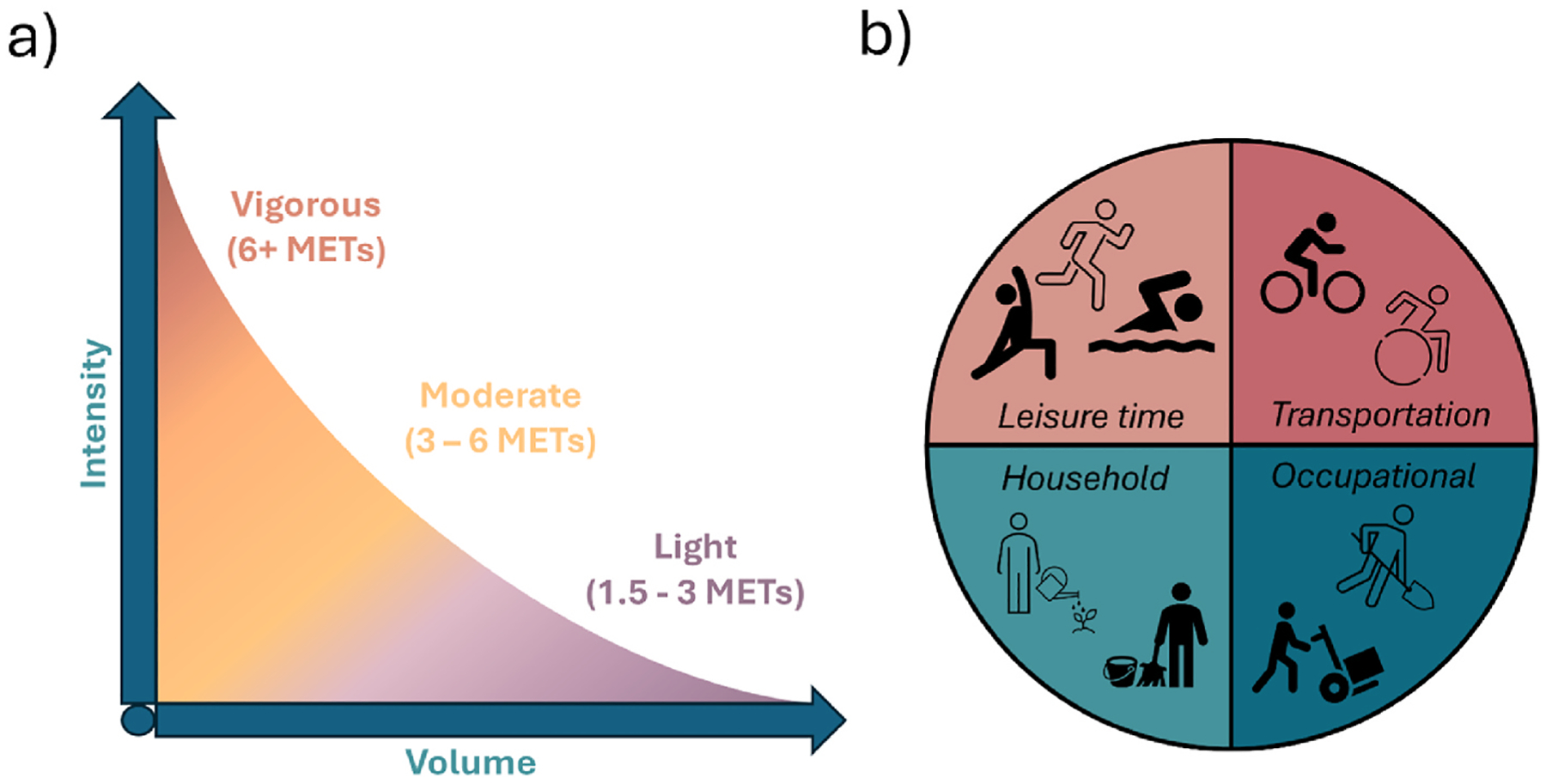
a) Physical activity intensities, and their interaction with volume for comparable health benefits. Metabolic equivalent of task (MET) represents a physiological measure expressing intensity of physical activity, with one MET being the energy equivalent expended by an individual while seated at rest. b) Physical activity domains.

Traditionally, physical activity has been classified using different intensity levels (light, moderate, vigorous) (Fig. 4a). The predominant and most reliable evidence of associations with health outcomes is available for regular moderate-to-vigorous physical activity (i.e., brisk walking, jogging and running) resulting in the WHO recommending adults accumulate 150–300 min of moderate-intensity or 75–150 min of vigorous-intensity physical activity per week to achieve health benefits (WHO, 2020). Conversely, there are currently no WHO recommendations for light-intensity physical activity (e.g., casual walking, light household work), due to comparatively fewer studies, despite the fact that light intensity activity is the main driver of total volume of activity energy expenditure (Lindsay et al., 2019) in the general population. Evidence from experimental and observational studies does however demonstrate associations between light-intensity physical activity and improved cardiometabolic health and reduced mortality risk (Chastin et al., 2019; Ku et al., 2020). Studies employing device-based methods can more accurately reflect low-intensity activities that are difficult to accurately capture using self-reporting methods. Despite the absence of WHO recommendations, light-intensity physical activity presents an opportunity for public health promotion. While more light-intensity physical activity may be required than moderate to vigorous physical activity to lower risk (e.g., 3–4 times higher to reduce risk of premature death (Ekelund et al., 2024a)), light-intensity physical activity is more pervasive (Young et al., 2016) and likely more achievable than moderate-to-vigorous physical activity (Ross et al., 2024).

Physical activity is defined by different domains, typically categorised as leisure-time, occupational, transport or household physical activity (Fig. 4b). Most research typically focuses on leisure-time physical activity (PAGAC, 2018; Beenackers et al., 2012) which observes consistent health benefit. For other domains, the evidence is mixed (Quinn and Gibbs 2023), with largely unknown impacts for household physical activity, and even adverse health effects observed for occupational physical activity (Holtermann et al., 2018). There is also evidence suggesting effect modification by domain (Quinn and Gibbs 2023), and interactions between them (Prince et al., 2021). Additional studies to measure and examine domain-specific physical activity are needed, and more evidence is also needed on the importance of situational aspects of physical activity (e.g., type and location, and whether it is voluntary), which can be particularly important when considering health inequalities.

##### 2.1.5.2. Assessment methods (objective measurements of physical activity).

Physical activity recommendations are based almost entirely on observational studies using self-reported data (WHO, 2020), prone to both recall and social desirability biases often resulting in over-estimation of physical activity levels (Warren et al., 2010). This likely leads to an underestimation of the true association, as evidenced by stronger associations from studies using device-based measurements (Ekelund et al., 2019). Similarly, most observational studies rely on physical activity levels measured only at baseline, again likely underestimating the true association (Ekelund et al., 2024b). Evidence from a prospective cohort study of Taiwanese adults suggests that associations with all-cause and cardiovascular mortality are stronger when using repeated measures of physical activity (Martinez-Gomez et al., 2022).

Evidence from several large, well-powered studies is now available (e.g., Dempsey et al. (2022), with observed associations orders of magnitude stronger than those using self-report data (Ekelund et al., 2024a). With enhanced granularity (i.e., seconds) allowing short and sporadic bouts of activity to be captured, this evidence-base more accurately reflects the totality of physical activity. This enables a shift from traditionally operationalised and rigid classifications, towards more comprehensive measures including device-measured physical activity energy expenditure (e.g., Strain et al. (2020), or device-measured daily step count, which is more easily understood by the public, and supported as a viable metric for assessing associations between physical activity and health outcomes (PAGAC, 2018). These measures that capture the full spectrum of physical activity more closely align with WHO guidance that “*doing some physical activity is better than doing none*” (WHO, 2020) therefore, observational studies utilising device-based measures are likely to play a prominent role in the development of future physical activity recommendations (Ekelund et al., 2024b). Other methods for assessing physical activity and energy expenditure include, for example, the doubly labelled water method that is considered a gold-standard, or calorimetry approaches, though such methods are expensive and not used in large studies (Ndahimana and Kim, 2017).

The increasing availability of data from smartphones and wearables means device-based measures have the potential to be readily integrated into long-term observational studies. However, many key challenges in the use of device-based measures at the population level remain, including concerns around representative sampling and wear time, validity and reliability, and between-device compatibility (Strain et al., 2022). For example, device-based measures are likely to over-represent privileged populations (Ding and Ekelund 2024). As with traditional measures of physical activity, there is also a need for greater standardisation among the increasing number of consumer wearables providing physical activity measures, which could be achieved through, e.g., advanced harmonisation approaches (Pearce et al., 2020). There is also a need to acknowledge that estimates derived from self-report and device-based measures are ultimately based on conceptually inequivalent approaches (Strain et al., 2022; Welk et al., 2019; Troiano et al., 2014). Consequently, as they cannot be measured on the same scale, it is necessary to pair the right DRF with the right population prevalence measure for correct impact assessment. In order to inform future guidelines, there is an additional need for more device-based evidence for important health outcomes including type 2 diabetes, certain cancers, and mental health (Ekelund et al., 2024a).

Areas of future innovation include integration of biomechanical and physiological data, which may be facilitated by advancements in bioengineering (e.g., skin patches), and the integration of multiple sensors in multiple body locations (Martinez-Gomez et al., 2025). Multi-sensor approaches would allow for better differentiation of activity types and postures, enabling more precise assessments of physical activity compared to approaches based on only one measure. Importantly, it is also recognised that device-based measures may be further complemented by self-report data that may better capture important contextual information (e.g., differentiating leisure time or commuting), allowing for a more accurate total characterisation of physical activity (Martinez-Gomez et al., 2025).

##### 2.1.5.3. Confounding and effect modification (physical activity and environmental exposures).

Research has demonstrated how physical activity and air pollution may interact, with physical activity behaviour and health effects potentially moderated by air pollution exposure (Tainio et al., 2021). Evidence from HIAs generally demonstrates health benefits from physical activity outweighing any adverse effects of air pollution exposure (Giallouros et al., 2020; Tainio et al., 2016). In highly polluted areas however this evidence is less certain Lee et al., 2024). The interactions and synergies between physical activity and air pollution are complex, and further stratified evidence (e.g., by time of day, intensity, domain) is needed to increase understanding (Hahad et al., 2023). Research is also limited for other environmental exposures, such as excessive heat or cold that can impact the physiological responses of physical activity (Périard et al., 2021) and may further influence physical activity patterns (Bernard et al., 2021). Also, it has been suggested that performing physical activity in green and blue spaces brings additional health benefits (Yen et al., 2021). Further research is needed to better understand how other environmental exposures may interact with health benefits of physical activity.

### 2.2. Cross-cutting research needs

Here, we highlight several ongoing challenges and research needs relevant to all pathways and environmental epidemiology more broadly. We focus on multi-exposures and evidence synthesis, that represent two areas of cross-cutting research needs specifically discussed in the workshop, but also highlight several other important cross-cutting topics.

#### 2.2.1. Multi-exposures

The quantification of health impacts of multiple exposures is challenging due to limited ERFs from two or multi-exposure models. ERFs used in HIA are typically derived from single-exposure models. However, populations are simultaneously exposed to multiple stressors and there is growing interest in the investigation of combined effects, and a desire to shift towards a broader exposome paradigm throughout the life-course (Wild, 2005).

Environmental exposures are often correlated (e.g., greenness, air pollution, road traffic noise in Klompmaker et al., 2020)). For example, traffic-related air pollution (TRAP) represents a complex mixture of pollutants, often highly correlated, and it is difficult to assess whether epidemiological associations are due to the direct effects of one pollutant or another, or to the mixture (HEI, 2022). This collinearity (or multi-collinearity when considering multiple exposures) arises due to commonality of sources, such as road traffic, or even meteorological conditions (Gowers et al., 2020). In case of positive correlations and similar directional effects for two exposures, summing the health burden from single-exposure models leads to an overestimation, and is generally avoided in practice (Chen et al., 2024b).

Consequently, several methods and statistical approaches to capture the impact of multiple exposures have been developed (Stafoggia et al., 2017; Maitre et al., 2022), like the ‘cumulative risk estimate’ (Crouse et al., 2015) or ‘g-computation’ approaches (Keil et al., 2020). These were employed in epidemiological studies to assess associations of multiple environmental exposures on hypertension (Chen et al., 2024d), stroke incidence (de Bont et al., 2023) and all-cause mortality (Dimakopoulou et al., 2024; Nobile et al., 2024). More studies applied two- or multi-exposure models which adjusted for the confounding effect from the other exposures (Stafoggia et al., 2022; Klompmaker et al., 2019, 2021; Nieuwenhuijsen et al., 2018). Whilst applicable to multiple exposure types, the literature is more developed for air pollution.

A recent study by Chen et al. (2024b) explored how using two-pollutants models can adjust potential under or overestimation of health impacts of air pollutant mixtures. In their work, they found that in two-pollutant models, associations with all-cause mortality for both PM_2.5_ and NO_2_ were attenuated compared to single-pollutant models. To demonstrate implications for HIA, they further estimated population attributable fractions (PAFs) using hazard ratios derived from single- and two-pollutant models, finding the former to be 38.6 % larger than the latter (PAF = 0.079 vs. 0.057) (Chen et al., 2024b). They applied the coefficient difference method proposed by COMEAP (COMEAP, 2018), which consists of adjusting coefficients from single-exposure models using coefficient differences for PM_2.5_ and NO_2_ from the more limited evidence base of studies with single- and multi-exposure models. They calculated the average coefficient difference between single- and two-pollutant models for PM_2.5_ (0.017) and NO_2_ (0.007) and applied this to a more extensive evidence base from the WHO systematic reviews.

While an improvement over single-pollutant models, two- and more complex multi-pollutant models face several challenges (Gowers et al., 2020; Dominici et al., 2010) including: 1) lack of interaction term, 2) multi-collinearity, and 3) transfer of effect. Additionally, relative risks from two-pollutant models, as with single-pollutant models, may also be confounded by other pollutants not routinely assessed (e.g., ultrafine particles, VOCs) (COMEAP, 2018) owing to, e.g., methodological complexity of measurement or limited public health consensus, and therefore whether or not they confound other pollutants cannot yet be accurately assessed. The multi-exposure approach presents many complex challenges, however, it will have significant implications for how we quantify health risks, and how subsequent policies and regulatory standards are designed (Dominici et al., 2010). Future research can also apply the methodology presented by Chen et al. (2024b) to other environmental exposures and outcomes in HIA (Chen et al., 2025). However, applying this methodology or similar to multiple different environmental exposures is likely to introduce complexities related to high-dimensional data, compounding challenges including interaction and multi-collinearity, as well as additional challenges such as nonlinear effects and differing variable types and measurement errors (Yu et al., 2022).

#### 2.2.2. Evidence synthesis and assessment approaches

Systematic reviews and meta-analyses (SRMAs) are increasingly used to synthesise health-effect studies of environmental determinants (Whaley et al., 2016) providing comprehensive, transparent and reproducible summaries of available evidence that guides policy and public discourse (Cooke et al., 2023). However, whilst often considered the “gold standard” for evidence synthesis, the methodological rigour of SRMAs varies, and poorly conducted reviews are prevalent (Forastiere et al., 2024a; Menon et al., 2022; Sutton et al., 2021). Bridging gaps in methodological and quantitative synthesis approaches (Rigaud et al., 2024), and improving how this evidence is applied in practice (Cooke et al., 2023), is necessary to improve the policy relevance of this epidemiological evidence.

Several frameworks exist for evidence synthesis in environmental health, including OHAT and the Navigation Guide (OHAT, 2019; Woodruff and Sutton, 2014). In a critical interpretive synthesis of current SRMA approaches several domains necessary to guide methodological processes are identified (Senerth et al., 2024), including guidance on selection of appropriate qualitative and quantitative synthesis methods, risk of bias assessment, and on reporting methods and results. Notably there is consensus on the importance of risk of bias assessment which, in face of substantial heterogeneity in key concepts considered and the tools/domains available, should be tailored depending on, for example, intended application or study design. Several risk of bias tools to evaluate observational studies on environmental and occupational exposures exist, such as ROBINS-E (Higgins et al., 2024), however there is no consensus on a best approach in these settings (Steenland et al., 2025; Eick et al., 2020). Additionally, integrating broader, more ‘narrative’ assessment in current evidence synthesis and assessment frameworks could ensure greater alignment with features relevant to, and maximize what can be learned from, observational studies in environmental health. As discussed in a Health Effects Institute commentary, existing formal approaches (e.g., GRADE, OHAT) are more oriented towards evaluating the quality of evidence of studies entering a meta-analysis, and less so towards determining the strength and nature of an association (HEI, 2022; Boogaard et al., 2023). Additionally, they often do not include specific criteria relevant to environmental health studies (Forastiere et al., 2024a). Employing a complementary ‘narrative’ assessment may therefore capture some of the important and complex nuances that may be missed otherwise (Boogaard et al., 2023).

In a recent commentary (Shaffer et al., 2025), U.S. EPA scientists outline several practical suggestions to greater facilitate the integration of epidemiological analysis in HIA, and enhance the value of published research. These include several easily implementable reporting practices (e.g., including null and non-significant results, justifying transformations, providing results on original scale, and reporting detailed exposure distribution information) as well as additional modelling practices (e.g., modelling in the low-exposure range, exploring non-linearity, exploring the influence of confounders with a tiered approach). Further recommendations have been provided elsewhere (Nachman, 2011), particularly for air pollution (Forastiere et al., 2024b; Fann et al., 2011). With increasing methodological complexity of SRMA approaches, pairing concise plain-language summaries with technical documentation and engaging scientific advisors will encourage transparent translation of evidence for decision-makers.

Existing evidence assessment approaches may be better tailored for use in environmental epidemiology, such as the growing adaptation and application of the Navigation Guide methodology for assessing SRMAs (Loomis et al., 2022; Teixeira et al., 2021). Another notable approach is the Burden of Proof methodology developed by the Global Burden of Disease (GBD) (Zheng et al., 2022). It utilises a burden of proof risk function, a novel meta-analytical tool for estimating the level of risk closest to the null hypothesis that is consistent with the available data, and provides a consistent way to understand, evaluate, and summarise the cumulative strength of evidence (Zheng et al., 2022). The GBD meta-analytical approaches may differ from other more ‘conventional’ approaches. For example, the GBD method for estimating temperature-mortality ERFs does not employ distributed non-linear lag models to capture lagged temperature effects. Instead, they assume short-term effects to occur on the day of exposure, a more generalisable and computationally manageable approach suitable for their global assessments, but results in potentially conservative or underestimated burdens (Burkart et al., 2021). Similarly, the current GBD approach for establishing PM_2.5_-mortality ERFs integrates household air pollution (and previously active/secondhand smoking (Brauer et al., 2024)) to extrapolate risks at high exposure. This extends coverage to high-exposure regions with limited epidemiological evidence (e.g., many low- and middle-income countries), however introduces uncertainty (Shaffer et al., 2019).

Umbrella reviews, systematic collections and assessments of SRMAs, are being increasingly undertaken to assess, synthesise, and summarise evidence to better inform decision-making (Ioannidis, 2016; Aromataris et al., 2015). Their limitations however include a lack of standardised methods (Shi and Wallach, 2022), and inability to capture recent evidence absent from SRMAs, which may be overcome through inclusion of recent high-quality individual studies (i.e., an Umbrella + review (Engelmann et al., 2023)). Umbrella reviews also depend on the quality of existing evidence, risking higher-level propagation of errors and biases. Similarly, whether reviewing primary or secondary studies, it remains challenging to identify informative studies and most likely sources of bias (Brunekreef et al., 2024).

#### 2.2.3. Standardised definitions, methodologies, and analyses

Across all exposure pathways we observe a need for standardised definitions (previously discussed, for example, for greenspace), and for standardised methodologies and analyses to allow for meaningful syntheses and comparisons between studies. This should, however, be balanced to ensure that methodological advancement is not limited and provides contextually nuanced insights. Moreover, observing consistency of associations across a diversity of methods (i.e., triangulation) strengthens the confidence in the evidence (Lawlor et al., 2016).

More broadly, this will require greater data harmonisation and collaboration within the scientific community across international and interdisciplinary studies (Dyer et al., 2024a). There is also a need for more open data sources, allowing access to and exploration of how ERFs are constructed. These are available for, e.g., for physical activity (Garcia et al., 2023), heat (Masselot et al., 2023) and air pollution (IHME, 2024).

#### 2.2.4. Incorporation of objective measurements

Objective measurements obtained from personal/wearable sensors are routinely incorporated in physical activity epidemiology (discussed previously in detail) and are increasingly being utilised in environmental epidemiology, particularly for air pollution. New sensor technologies have immense opportunity for exposure and health outcome assessment, with benefits including enhanced individual-level exposure assessment at high spatiotemporal resolution, integration with behavioural and physiological data, and scope for scalable, low-cost data collation (Tonne et al., 2017). However, several considerable theoretical and methodological challenges hinder their wider application, requiring further technological development and research (Doherty et al., 2021).

Data accuracy remains a critical issue (particularly for low-cost sensors) which may result in substantial bias (Ueberham and Schlink, 2018). Consequently, personal sensors require careful assessment and validation prior to application in research (Khreis et al., 2022; Jerrett et al., 2017). Similarly, while GPS-enabled sensors provide valuable temporal data for health research (Kerr et al., 2011), they are often limited in their ability to determine indoor locations due to signal loss (Asaad and Maghdid 2022) limiting their application for investigation of indoor/outdoor exposure differences. Several other limitations, such as device reliability, participant burden, and meteorological interference, inhibit their wider uptake and application, and are discussed elsewhere (Doherty et al., 2021; Helbig et al., 2021).

#### 2.2.5. Consider mobility, and where exposure occurs

In most epidemiological studies, exposure is measured or modelled based on the participants’ residential address. However, this practice fails to fully account for the totality of exposure individuals experience (i.e., exposure misclassification, or measurement error), and thereby introduces uncertainty, potentially leading to biased risk estimates. This wider issue remains a topical area of active research across various environmental exposures (Katsouyanni and Evangelopoulos, 2022; Wei et al., 2022; Wilt et al., 2023). Here, we focus specifically on exposure misclassification relating to individual and population-level mobility, and indoor/outdoor differences.

Exposure estimates based on static measurements largely disregard individual daily mobility, leading to inconsistent or unreliable estimates of individual exposure (Kwan, 2012, 2018) across various environmental factors which depend on mobility (Cai and Kwan, 2024). At the population level however, as highlighted by recent publications which observed similar health effects using residential vs. dynamic exposure assessment of air pollution exposure, the overall bias in epidemiological studies may be small (Hoek et al., 2024; Ntarladima et al., 2021). A large census-based cohort study in Canada reported nearly identical associations with both residential and time-weighted average exposure of residential and work address (Christidis et al., 2021) but such associations may be context-specific. deSouza et al. (2024) found in a health impact study slightly smaller estimates of annual mortality attributable to NO_2_ (−2 %) and PM_2.5_ (−0.3 %) when deriving exposure levels based on home, instead of home and workplace locations. More research is necessary to understand the extent residential-based exposure assessment leads to bias for other mobility-dependent environmental exposures, not just air pollution for which there is more information (Hoek et al., 2024).

Additionally, in recognising that individuals typically spend around 80–90 % of their time indoors, there is a need to further consider differences between indoor and outdoor exposures. Temperature-related research rarely incorporates indoor environmental conditions that can greatly differ from outdoor environments (Nazarian and Lee, 2021), as demonstrated in a global study identifying weak-to-moderate relationships between indoor and outdoor temperatures (Hou et al., 2023). This relationship may vary based on ambient conditions (i.e., seasonality), and different epidemiological associations may be observed based on this exposure misclassification (Xia et al., 2024). For transport noise, some studies provide attenuation factors for indoor levels at the population level (Foraster et al., 2014; Locher et al., 2018); however, these can be difficult to apply as they depend on building characteristics and personal behaviours (e.g., window opening), as well as underlying exposure-effect pathways (direct vs. indirect). Even for air pollution, where comparatively more research has been undertaken, many knowledge gaps remain, complicated by the multitude of indoor environments (e.g., homes, schools) and variety of sources therein, as well as personal behaviours that may influence them (e.g., ventilation) (Vilcins et al., 2024a,b). There is also a need to consider factors that may influence the effect of indoor-outdoor relationships such as housing quality (Ferguson et al., 2021).

Future epidemiological research could further integrate studies that account for exposure in other microenvironments, such as in transport or indoor environments (Smith et al., 2016; Ferguson et al., 2023). This may be actualised by capitalising on personal/wearable technologies and smartphone-based mobility data that capture not only what individuals are exposed to, but where this exposure occurs (Birenboim et al., 2021). Other opportunities include microsimulation and agent-based modelling that can characterise individual behaviours and exposures for whole populations with high spatiotemporal and sociodemographic resolution (Staves et al., 2023).

#### 2.2.6. Spatial resolution of analysis

Epidemiological studies exhibit sensitivity to the spatial scale of exposure estimates and in practice, the use of finer spatial resolution is thought to result in a higher effect size and smaller bias (Wei et al., 2022; Vodonos et al., 2018). For example, in a CanCHEC (Canadian Census Health and Environment Cohort) study researchers observed stronger associations between air pollutants and lung cancer and respiratory mortality at smaller spatial scales (e.g., 1 km × 1 km, vs. 10 km × 10 km) (Crouse et al., 2020). Non-accidental and cardiovascular mortality differed less with spatial scale. For noise, as demonstrated in a Swiss study on myocardial infarction mortality, higher resolution estimates (e.g., façade estimates and fine scale noise maps (10 × 10 m)) are preferred to coarser scale estimates which can introduce more bias and attenuate health effect estimates (Vienneau et al., 2019). Similarly for greenspace, map resolution and spatial scale should be considered and may explain observed heterogeneities (Wang et al., 2024).

Relatedly, spatial resolution should be considered when estimating health burdens and impacts. Several air pollution studies have demonstrated how exposure and mortality estimates are sensitive to the spatial resolution of exposure maps (Korhonen et al., 2019; Fenech et al., 2018). In a recent HIA study in Colorado, USA, deSouza et al. (2024) demonstrated that the use of county-level NO_2_ and PM_2.5_ estimates, compared to remotely sensed 1 km × 1 km resolution estimates, resulted in a 50 % and 10 % decrease in attributable mortality, respectively, related to fine-spatial resolution variability of air pollution exposure. The spatial resolution of health data should also be considered in HIAs (Jaikumar et al., 2025). For example, in the U.S. deSouza et al. (2024) demonstrated the use of block-level baseline mortality rates, instead of county-level data, yielded a 10 % higher estimated annual mortality attributable to PM_2.5_ and NO_2_. In India, Chowdhury and Dey (2016) similarly demonstrated that the use of a uniform nationwide baseline mortality rate dataset, instead of state-specific baseline mortality rates, resulted in ~15 % lower PM_2.5_ attributable premature mortality estimates. Increased efforts to provide high-resolution exposure, demographic and health data are required, and researchers should prioritize exploring associations at multiple spatial scales (Dyer et al., 2024a; Dyer et al., 2024b; Jaikumar et al., 2025). Moreover, incorporating finer-scale spatial exposure data can help identify localised hotspots, and uncover environmental inequalities, allowing for prioritisation of local interventions (Mitsakou et al., 2019). Spatial scale should also be considered when examining multiple exposures, as these may be measured or modelled with varying precisions, potentially leading to a transfer of effect.

#### 2.2.7. Evidence for morbidity outcomes

Traditionally, with the exception of noise, HIA studies have focussed more on mortality impacts than morbidity. For example, air pollution HIAs have largely focussed on all-cause and cause-specific mortality, based on ERFs synthesised from long-term epidemiological evidence (Forastiere et al., 2024b). For some health determinants, this may reflect a lack of epidemiological evidence for morbidity. For example, there are comparatively fewer studies for temperature-related morbidity (Cheng et al., 2019), and while there is evidence for some morbidity outcomes (e.g., emergency hospital admissions), the evidence-base is stronger and more robust for all-cause mortality, with mortality data being more readily accessible (UK Health Security Agency, 2024). Morbidity has significant negative societal and economic impacts, through both direct and indirect costs of illness, including medical expenditures, rehabilitation costs, informal care, and labour productivity losses, and individual loss of utility or welfare cost (OECD, 2019). Morbidity ERFs could therefore allow for more comprehensive assessment of the cost of environmental stressors to inform policy analysis (Ru et al., 2023).

Additionally, there is also a need to consider multimorbidity (the co-occurrence of two or more health conditions in an individual) that impacts a substantial, and likely growing, proportion of the population. Environmental determinants are associated with increased risk for several chronic conditions (such as diseases of the circulatory and respiratory systems, neurological disorders like dementia, and metabolic outcomes like diabetes), and through their accumulation, may be associated with multimorbidity. Research has demonstrated associations between air pollution and multimorbidity, suggesting certain organ systems may be more vulnerable than others (Ronaldson et al., 2022). The evidence base however is sparse, and multifaceted and complex by nature (The Academy of Medical Sciences, 2018), but due to its substantial economic and societal burden (Tran et al., 2022) warrants greater consideration.

#### 2.2.8. Evidence from population sub-groups

Across all exposure pathways, there remains a paucity of sub-group ERFs, thereby meaning HIAs cannot produce stratified estimates, precluding equity-informed policy recommendations (Dyer et al., 2024b). More evidence is needed for specific sub-groups (e.g., stratified by age, sex, race/ethnicity, socioeconomic status, and those with pre-existing health conditions) who may be more susceptible and/or vulnerable to adverse health effects. For example, those of lower socio-economic status (SES) are more likely (though not consistently) to be exposed to higher levels of air pollution, and generally, stronger associations between air pollution and health outcomes are observed (Hajat et al., 2021). Similarly, stronger associations for heat-related mortality and morbidity are found for the elderly, people experiencing homelessness, and low SES groups (Benmarhnia et al., 2015; Noor et al., 2025).

The implications of sub-group ERFs for health estimates have been highlighted. For air pollution, recent analyses from deSouza et al. (2024) showed the use of racial/ethnic-specific CRFs in place of an overall CRF in the U.S. resulted in estimates of mortality attributable to NO_2_ differing by as much as a factor of 2.9. Other research in the U.S. has also reported steeper ERFs for PM_2.5_ and mortality for Black persons than White persons (regardless of income) (Josey et al., 2023). However, a systematic assessment of findings across evidence-base is lacking. For temperature-mortality associations, a global study observed significant variations by age, with the oldest age groups almost universally observing higher heat and cold risks/burdens than the youngest (Scovronick et al., 2024). Producing reliable estimates for sub-group specific CRFs, and to explain opportunities for transferability, caveats and limitations, however, requires very large populations to reliably estimate them.

Additionally, research should also assess differences between urban and rural populations. For example, while comprehensive temperature-related mortality assessments are available at the local-area (Gasparrini et al., 2022) and regional scale (Masselot et al., 2023), they are generally restricted to urban populations that typically experience higher levels of temperature stress due to the urban heat island effect (Manoli et al., 2019). Evidence from rural areas is largely still missing, and the existing evidence reveals conflicting results with regard to the effect of urbanisation on heat vulnerability which may relate to a number of factors including access to health care services, greenspace, type and quality of housing, lifestyles and cultural differences (Lee et al., 2022; López-Bueno et al., 2022). Further investigation of urban/rural differences may also help reveal important contextual factors and drivers of health (for example, considering the ‘paradox’ of high greenspace and poor health in rural Central Appalachian communities (Dong et al., 2024)) that should be considered to ensure targeted interventions achieve expected benefits.

#### 2.2.9. Evidence from low- and middle-income countries

There remains a lack of epidemiological evidence from many low- and middle-income countries (Rogowski et al., 2025; Brauer et al., 2024). Expansive regions including Africa, the Middle East, and South Asia remain poorly represented (Health Effects Institute, 2022; Gasparrini et al., 2024). Expanding research to other regions could help elucidate largely unknown drivers of geographical heterogeneity. Additionally, low- and middle-income countries frequently experience higher levels of harmful environmental exposures, and current epidemiological evidence is often lacking at these higher exposure ranges. To enable research in low- and middle-income countries, there is a need for enhanced and practical exposure assessment, for which low-cost sensors, remote sensing, or land-use regerssion models may be well-suited (Clark et al., 2022; Brum et al., 2024). Additionally, there is a need for more longitudinal studies in low- and middle-income countries (Chen et al., 2023c; Rojas-Rueda et al., 2021). This could be facilitated through better collaboration and development of multinational studies which through mutual contribution, method and data sharing, can advance epidemiology at a global scale (Gasparrini et al., 2024). It will be essential for funders to expand their reach to these regions, which should be done in a way that acknowledges imbalances of power and resources (Charani et al., 2022).

Common research practices such as limiting literature searches to articles published in English, further contributes to a loss of available knowledge from low- and middle-income countries, and may ultimately bias effect estimates in meta-analyses (Neimann Rasmussen and Montgomery, 2018). Review teams should therefore consider resource translation services or collaborative screening when language skills are limited.

By strengthening research capacities in low- and middle-income countries, we may unravel unexplained heterogeneities present across epidemiological studies and better understand risk over global exposure ranges. This process involves building both individual and institutional research capacities in these settings, which relies not just on provision of funding but in fostering research culture and local ownership (Malekzadeh et al., 2020). This capacity building process must also acknowledge and navigate prevailing power asymmetries in the global health research system, and seek, through decolonisation of research practices, to foster more equity-oriented approaches (Kumar et al., 2024).

#### 2.2.10. Generalisability of evidence

An ERF typically synthesises available evidence into a single global estimate, derived from a wide range of participants drawn from various study settings. Applying a single global ERF in a HIA provides several advantages: reduced uncertainty by pooling data from multiple sources, streamlined application while enabling greater comparability, and providing a coherent basis for developing broad polices and recommendations (Forastiere et al., 2024b). Consequently, global ERFs may be recommended for quantitative HIA (COMEAP, 2022a), however in practice, this decision is much more nuanced.

The generalisation of an ERF, derived from one population or context, may introduce systematic biases when applied to another setting which may not share the same characteristics. This issue is particularly acute given that most epidemiological evidence originates from high-income countries, notably Western Europe and North America. For example, ERFs for heat-related risk are primarily derived from studies of temperature regions that are generally high-income with better access to adaptive measures, which may not be representative of low- and middle-income countries in the tropics with higher temperatures and less adaptive capacity (Green et al., 2019). Between different populations, several factors such as susceptibility, baseline health status, diet, or genetic background may differ and influence health risks (Singh et al., 2024; Eisen et al., 2024; Lim et al., 2019; COMEAP, 2025). Relatedly, such factors may also influence each other, as well as influencing both risk and underlying health, introducing further complexity requiring more advanced statistical methods (Lloyd et al., 2023). In place of a single global ERF, a regionally (or locally) derived ERF may be employed that may better represent local population characteristics or environmental conditions, and therefore may be better suited for HIA studies targeting specific populations or regions (Pascal et al., 2016). Ultimately, the decision on whether to employ a global or regional ERF will depend on several factors such as the nature of the assessment, the specific policy question, and the intended application (Forastiere et al., 2024b), as well as the actual presence of regional heterogeneity between summary effect estimates, if known (Lee et al., 2023).

In some cases, ERFs derived from a relevant population may not be available. In many low- and middle-income countries, epidemiological evidence is often scarce, meaning HIA studies must rely on ERFs derived from, primarily, high-income settings resulting in uncertainties in estimated health impacts (Thondoo et al., 2022). Accordingly, there is a need for further research and guidance on the adaptation of epidemiological evidence to different contexts, specifically when epidemiological evidence is presently unavailable. Future research should explore how advanced statistical methods (e.g., Bayesian models) may be utilised to adjust estimates to these contexts and, when applied, uncertainty estimates should be provided (Nethery and Dominici 2019).

Evidence generalisability remains an active area of research (Lesko et al., 2017), encompassing other aspects not discussed here, such as extrapolating estimates beyond observed exposure data (Nethery and Dominici 2019), and non-linearity (Cox, 2020), and requires further consideration to reduce uncertainties introduced.

#### 2.2.11. Temporal validity of evidence

The temporal validity of derived ERFs (i.e., how long published results be expected to remain valid and applicable to HIA) remains a critical issue. Since ERFs from SRMAs represent a snapshot of the available evidence at the time searches were conducted, confidence in their findings may degrade over time as new evidence emerges. For instance, clinical evidence from a report assessing 100 systematic reviews indicated that 23 % of reviews were ‘out of date’ (i.e., substantively changed conclusions relating to statistical significance or effect magnitude) within two years, and 7 % were already outdated at time of publication (Shojania et al., 2007). This however may not hold true in all contexts, and while new studies can always be expected, the emergence of new evidence may not translate to a change in ERFs, particularly when there is already a large body of evidence like for air pollution. Further investigation into the temporal validity of published ERFs in an environmental health setting is therefore warranted, and from this, methodological guidance on if, when, and how evidence syntheses should be updated may be further developed. Existing guidance from the Cochrane Collaboration on systematic reviews of interventions, for example, states all Cochrane Reviews should be periodically assessed to determine whether an update is necessary, for which a decision framework for deciding when and how it should be updated is also available (although a general guideline of every two years is frequently cited) (Cumpston and Flemyng, 2024). Other relevant research directions include the viability, and practicality, of “living” systematic reviews that are continually updated to incorporate new evidence as it emerges (Elliott et al., 2017). Living systematic reviews are particularly well suited when:1) the question is a priority for decision-making, 2) the certainty in existing evidence is low and therefore, new evidence is likely to influence current findings, and 3) new emerging evidence is likely (Elliott et al., 2017). Additionally relevant is the concept of an “exit” meta-analysis, that would signal no further research is needed once a meta-analysis has conclusively addressed a research question (Abdulmajeed et al., 2025).

Temporal validity becomes particularly pertinent when considering the world’s rapidly changing exposure patterns. Already, for example, we have observed the substantial impacts of regulatory and technological interventions on global air pollution emissions (Oreggioni et al., 2022) resulting in changes to the TRAP mixture. Notably, climate change is expected to impact future temperature-related risks (Masselot et al., 2025; UK Health Security Agency, 2024). Elevated temperatures may also influence the vegetation composition of urban greenspaces and the extent to which they confer benefits due to air pollution mitigation (Allen et al., 2010). Elevated temperatures can also accelerate photochemical reactions resulting in higher O_3_ concentrations in urban areas and increased biogenic VOC emissions (Chang et al., 2023). Additionally, due to increasing wildfire emissions, climate change is further resulting in changes in air pollution composition (Xu et al., 2023). In order to better understand the temporal validity of current ERFs, it will be necessary to consider changing exposure patterns, co-exposures and modifying factors (Vanos et al., 2020; Zhao et al., 2025).

### 2.3. Implications for quantitative health impact assessment and policy

**Fig. 5.**
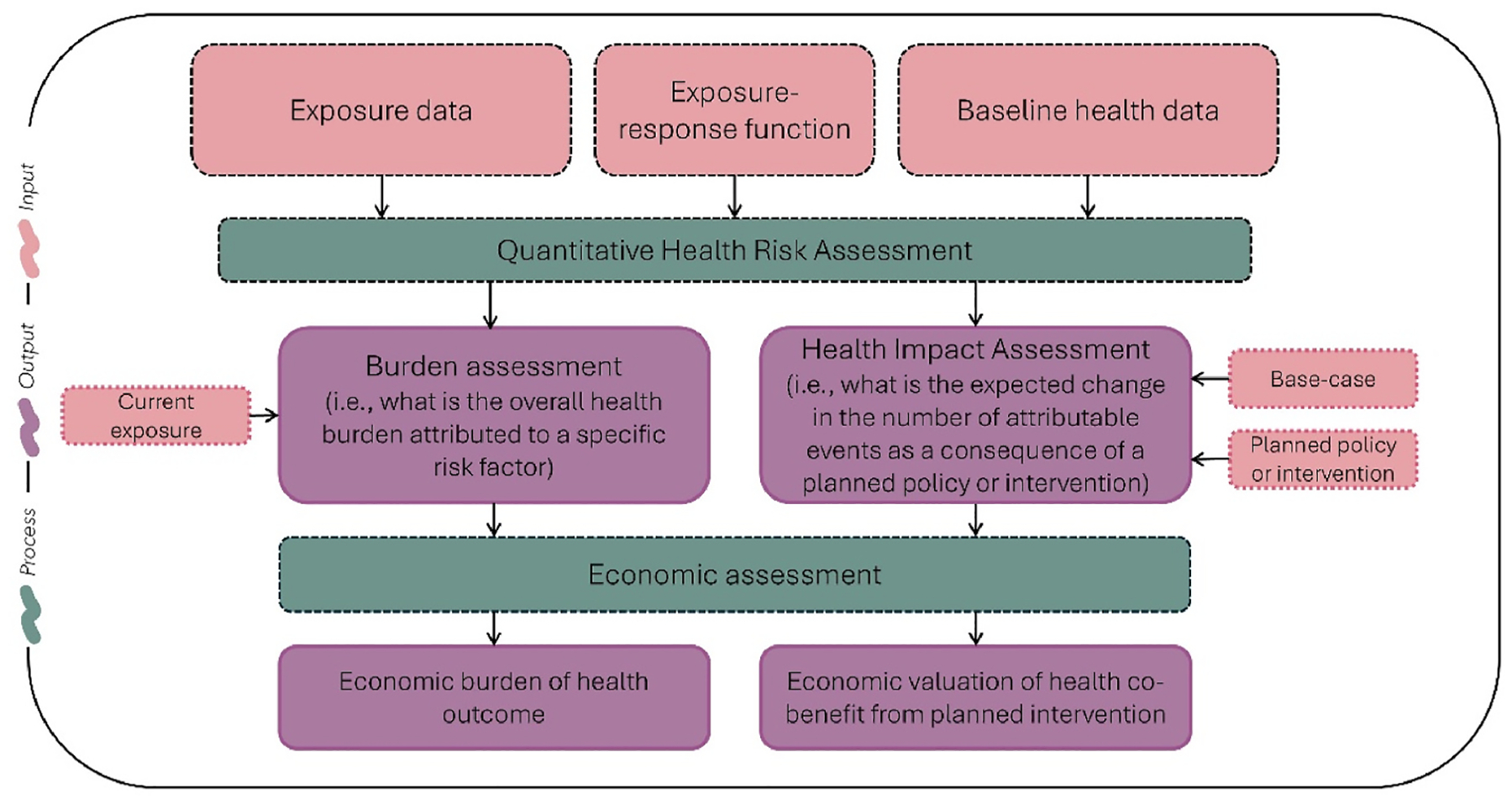
Simplified schematic of quantitative health risk assessment processes

HIA, or health impact modelling, enables quantitative understanding of the impacts of urban planning and transport scenarios on population health (Nieuwenhuijsen et al., 2019). The basic quantitative HIA process is well-established (WHO, 2016), and typically involves the use of exposure data and baseline health data, combined with ERFs to quantify health effects (Fig. 5). By comparing the health burden of counterfactual exposures to environmental stressors (e.g. air pollution, noise) and behaviours (e.g. physical activity) attributable to a policy or hypothetical scenarios, these assessments are intended to support evidence-informed decision-making. Quantitative risk assessment approaches play a central role in advancing public health and policy. For example, quantitative HIA has played a crucial role in the formulation of air quality guidelines and regulatory criteria in Europe and the U.S. (Forastiere et al., 2024b), and burden of disease approaches like the GBD have provided substantial contribution to the development of global health policy (Murray, 2022). In this section, we further explore the implications of applying ERFs in the HIA process, and present several directions that could lead to more impactful assessments.

#### 2.3.1. ERFs in the HIA process

ERFs are key inputs in quantitative HIAs (Fig. 5), but are often a substantial source of uncertainty, leading to heterogeneity in health impacts (Khomenko et al., 2021; Barboza et al., 2021; Castro et al., 2022; Sohrabi and Khreis, 2020; Malmqvist et al., 2018). In a review of global estimates of mortality attributable to air pollution (Pozzer et al., 2023), the choice of ERF was found to be the most significant factor of disparity between studies. Most evidence comparing uncertainties across different HIAs is available for air pollution. Future studies should explore how HIAs of different environmental health determinants respond to different model inputs. Other inputs and methodological choices associated with differences in estimates include differences in 1) exposure scales, 2) demographic data, and 3) assumptions in exposure range, as well as 4) the choice of counterfactual scenario (Pozzer et al., 2023; EEA, 2022). Importantly, uncertainty persists throughout all phases of quantitative health risk assessment. Differing sources and characteristics of these uncertainties have been described by different frameworks (Knol et al., 2009; Briggs et al., 2009), and may further interact in different ways in their contribution to overall uncertainty (Pozzer et al., 2023). Value of Information approaches can assess where to best invest in filling data and input gaps by mapping impacts of various input parameters on total uncertainty (Schroeder et al., 2025).

#### 2.3.2. Selecting ERFs for application in HIA

ERFs for HIA application are frequently drawn from existing literature (Benavides et al., 2022). Selecting an ERF from the multiplicity of published SRMAs however remains an ongoing challenge (Rigaud et al., 2024), with a growing, heterogeneous body of evidence leading to uncertainty around selecting the most accurate or appropriate ERFs to apply in HIA (Dyer et al., 2024b). For example, city- and age-specific temperature-mortality ERFs developed by Masselot et al. (2023) represent the best available evidence, utilised in several HIA studies (Lung-man et al., 2025; Khomenko et al., 2025). Similarly, global-scale studies often employ GBD-derived ERFs which apply consistent methodological approaches with explicit uncertainty quantification, and comprehensive integration techniques to ensure global exposure coverage. The ERF selection process is nuanced, depending on factors including research question and intended purpose, evidence availability, or policy considerations. Accordingly, the process should be proportionate to the scale of anticipated impact, in recognising that not all decisions require the same granularity.

Forastiere et al. (2024a), in a specific attempt to overcome umbrella review limitations, determined reliable ERFs for morbidity outcomes to apply in a HIA on long-term air pollution exposure. Their approach first uses causality determinations provided in the U.S. EPA’s ISAs as the basis for pollutant-outcome pair selection. Thus ISAs, representing the most authoritative causal assessment based on comprehensive synthesis and evaluation of available evidence, are leveraged to underpin selection of pollutant-outcome pairs with the most robust scientific evidence available. Then, they undertook a systematic literature search to identify SRMAs of relevant morbidity outcomes, which were then appraised to select those regarded as credible sources of ERFs for use in HIA. If necessary, any identified errors were corrected, and an updated meta-analysis was undertaken. Finally, they provide a classification of outcomes to provide recommendations on the reliability of quantification if applied in HIA. Several ERFs related to the incidence (onset) of disease associated with long-term exposure to PM_2.5_ and NO_2_ were proposed and, the study provided information regarding the range of mean exposures for which the uncertainty of a risk assessment is minimised.

**Fig. 6.**
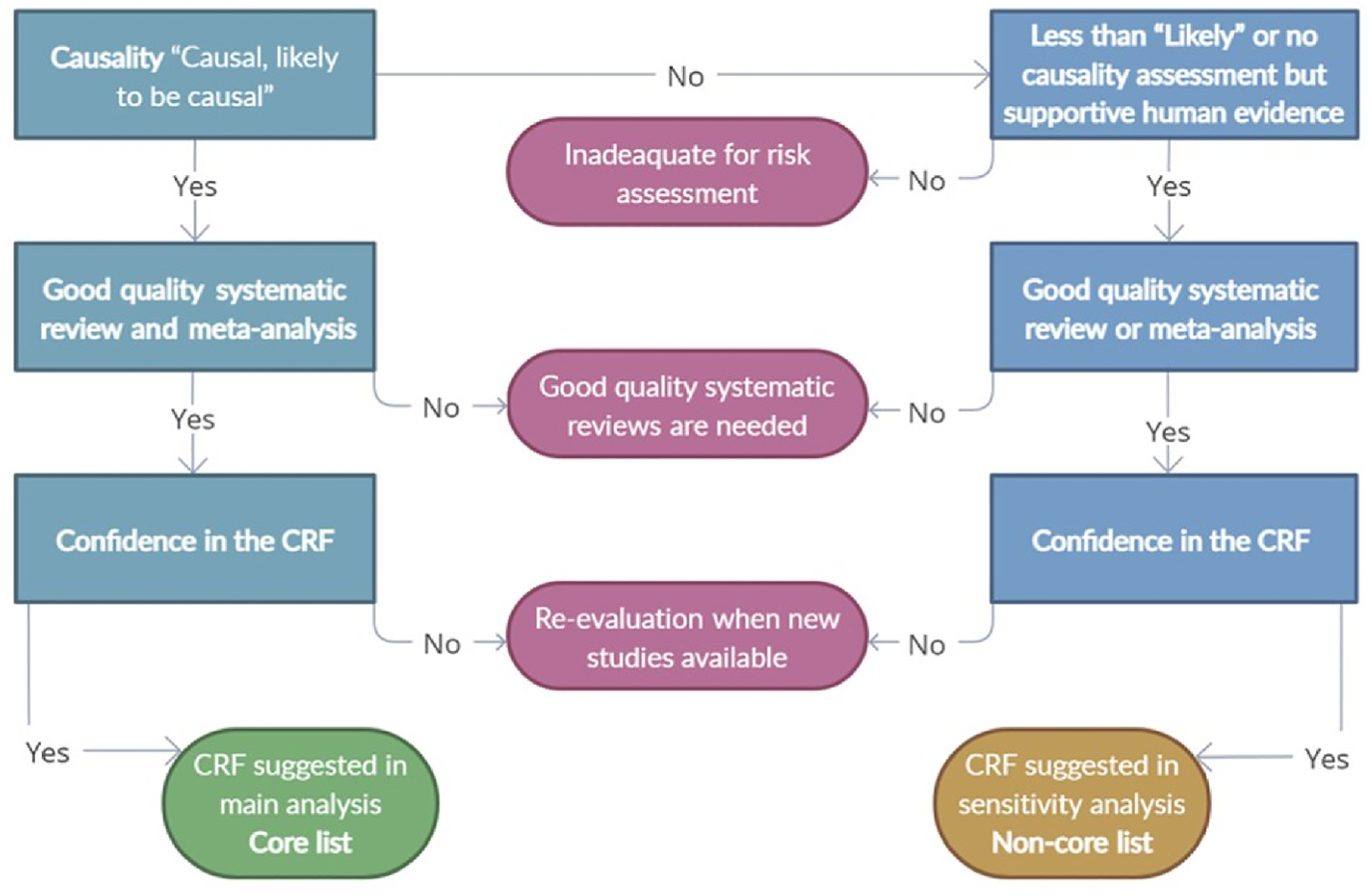
Schematic representation of the steps in choosing the appropriate concentration-response function (CRF). Figure from Forastiere et al. (2024b), licensed under CC BY-NC-ND 4.0 (http://creativecommons.org/licenses/by-nc-nd/4.0/).

Subsequently, Forastiere et al. (2024b) proposed a structured and comprehensive framework for selecting an appropriate ERF (Fig. 6). In this framework, causality determination, methodological quality, and the confidence in the evidence all contribute towards the selection process. However, while we must be certain that a health determinant is causally related to a specific outcome before confidently quantifying its impact, relying solely on ISAs as basis for inclusion may be a conservative approach. Limitations of using ISAs include their currency (e.g., the last assessment for NO_2_ was conducted in 2016) that risks omission of emerging hazards (i.e., recently identified determinants or effects) (Rigaud et al., 2024). This framework has since been applied, for example, to analyse the scientific evidence of health risks related to chemicals included in the EU Ambient Air Quality Directive (AAQD) (Orru et al., 2025).

#### 2.3.3. Improving the HIA process and its impact

Ultimately, uncertainties in the HIA process complicate the communication of environmental health risks to policymakers and the public. Crucially, such uncertainties are inherent in all parts of the policy-making process and not HIA; nevertheless the acknowledgement and quantification of this uncertainty, which should be proportionate to impact and complexity, serves to better support policy-making (Azzini et al., 2020). While there are existing resources to provide guidance and best-practice in conducting HIA, e.g. from WHO (WHO, 2025) or U.S. EPA (US EPA, 2017), there is a pressing need for better, more coordinated resources for improving HIA processes. In addition, increasing fluency of environmental epidemiologists in HIA processes, and vice versa, could facilitate greater consideration of data needs when planning and presenting research (Shaffer et al., 2025). Creating a common understanding among producers and users is essential in avoiding misinterpretation, promoting data collection, and addressing knowledge gaps.

Additionally, we note that the HIA process could more effectively contribute to policy development by integrating health evidence with wider socio-economic considerations. While an HIA can assess physical health impacts associated with environmental stressors, it typically does not account for their broader socio-economic costs, such as the cost of illness, lost productivity, well-being or reduced welfare. Because policy decisions often require cost justification and must balance pragmatism, feasibility, and social acceptability, there is a need for stronger recognition of, and integration between, health impact evidence and socio-economic valuation. Reconciling these outputs would ideally guide the design of more comprehensive and effective policies for improving population health.

Similarly, while a HIA can provide evidence in support of policy or hypothetical scenarios, there remains a need to evaluate the health and equity impacts of policies and interventions more broadly (Garber et al., 2024). This should be conducted at all stages of the policy process (Benavides et al., 2022) and there are methods (e.g., synthetic control methods, difference-in-difference) which can provide valuable evidence on the longitudinal effects of measures accounting for challenges (for example, the frequent co-occurrence of policy measures) (Garber et al., 2024). In order to monitor policy implementation more effectively, measurable policy targets, that are often absent, are also required (Lowe et al., 2022).

Finally, we acknowledge that our focus lies solely on the quantitative aspect of HIA. However, in order to provide a comprehensive assessment, it is essential to also incorporate qualitative and participatory aspects of HIA (Nieuwenhuijsen et al., 2017).

## 3. Conclusion

We have provided an expert-guided, in-depth discussion focussed on exposure-response functions and their application to quantitative health risk assessment. We critically synthesise and assess a large, disparate and diverse body of literature, mapping knowledge gaps and proposing specific areas for further research. Crucially, we show that the estimation, selection, and application of ERFs is highly nuanced and context-dependent, underscoring the need for critical assessment of their application and more systematic and transparent approaches to strengthen their utility for policy-making. The proposed research agenda is intended to accelerate the translation of scientific knowledge into actionable strategies that support healthier and more sustainable cities through urban and transport planning. Our recommendations draw from an international workshop that convened experts and stakeholders across disciplines, highlighting the importance of continued interdisciplinary collaboration. We encourage similar activities to provide a forum for generating new research ideas, advancing methods, and fostering knowledge exchange across sectors and geographies. Importantly, future efforts must prioritize equity, including participation and perspectives from low- and middle-income countries.

## Figures and Tables

**Table 1:** Relevant definitions of exposure-response, concentration-response and dose-response functions.

Term	Definition	Uses
Exposure-response function (ERF)	ERFs relate a health outcome to the level of exposure to a specific health determinant. Exposure in this context can represent the total contact, as a product of intensity, duration and frequency to account for when, where, and how much exposure occurs.	*air pollution, noise, non-optimal temperature, greenspace*
Concentration-response function (CRF)	CRFs relate a health outcome to the concentration of a health determinant in the environment (often employed as a surrogate for exposure).	*air pollution*
Dose-response function (DRF)	DRFs relate a health outcome to the dose* of a health determinant received. * *For air pollution, the term dose commonly represents the amount of air pollution inhaled by subjects or participants, as a product of pollutant exposure and minute ventilation*. * *For physical activity, the term dose represents the amount of physical activity performed by subjects or participants and therefore, the DRF refers to the relationship between activity dose and human health outcomes*. * *For greenspace, the term dose usually represents the amount of greenspace experienced by subjects or participants, reflecting contact with and use of greenspace and differing from proximity to greenspace*.	*air pollution, physical activity, greenspace*

**Table 2:** Summary of open research questions and proposed research directions based on pathway-specific discussion of research needs.

Pathway	Open Research Questions and Proposed Research Directions
Air pollution	Are recently observed associations between PM_2.5_ and mortality stronger? If so, why? What factors influence the shape of the PM_2.5_ exposure-response curve? Does it vary by region? Are certain components of PM_2.5_ more harmful? And to what extent do the reported associations represent a causal effect, or the effects caused by other correlated pollutants? And what will be the effect of changing compositions including in the context of a changing climate? What are the health effects of air pollutant mixtures? And how can we measure the extent to which associations reported for one pollutant represent a causal effect, and to what extent do they represent effects caused by other correlated pollutants? Do gaseous pollutants (O_3_, NO_2_) modify PM_2·5_ toxicity? Are their effects additive, synergistic, or antagonistic? How do different ERF shapes (e.g., linear, supra-linear, sub-linear) impact HIA, especially at low-to-moderate concentrations? What new statistical approaches can robustly resolve multipollutant exposures, incorporate omics data, and support integration with experimental toxicology?
Noise	How do different noise exposure-estimation methods influence observed effects? Are different sources of noise associated with different effects? And in what circumstances is it suitable to use pooled (e.g. transport) estimates in lieu of source-specific estimates (e.g. construction)? How do noise sources and characteristics vary by setting? How generalisable is the current evidence base, that is largely derived from European studies? What scalable methods (e.g., land-use regression) can be applied to develop fit-for-purpose noise exposure maps especially in low- and middle-income countries? How do multiple noise sources interact in affecting health? What is the relationship between transportation noise and air pollution? How best can studies account for potential effect transfer?
Greenspace	How can we better define greenspace, and what metrics or measurements best represent specific outcomes or pathways? How do different mechanistic pathways, or contexts, influence specific health outcomes? How do spatial resolution and measurement choice influence ERFs, and what aligns best with specific mechanistic pathways? What is the evidence for other landscapes, or nature characteristics (e.g., biodiversity, naturalness), and how can these be better captured and incorporated in research? To what extent do subjective perceptions versus objective greenness metrics differentially affect health outcomes? How do culturally and contextually relevant perceptions modify greenspace impacts? What mediators (e.g., physical activity, social cohesion, stress reduction) and moderators (e.g., socioeconomic status, age, gender) affect greenspace–health associations? How does seasonality or temporal exposure (e.g., summer peaks) influence greenspace-associated benefits?
Non-optimal temperature	How do populations adapt to non-optimal temperatures, and can long-term effects be disentangled from this? How do health risks associated with non-optimal temperatures vary across different climates, geographies, and population subgroups? How do we account for climate change in current modelling approaches, to capture processes such as population adaptation, and incorporate elements of climate justice? How does temperature interact with other environmental exposures and modify their health effects? What are the long-term health effects of chronic exposure to non-optimal temperatures? What are the best practices for projecting future temperature-related health burdens under varying climate, demographic, and adaptation scenarios?
Physical activity	How do associations of physical activity vary by intensities or domains not routinely captured? How can objective measurements best capture physical activity associations, and how comparable are these results to traditional approaches? What are the trade-offs of using device-based versus self-reported measures, and how should we align dose-response functions (DRFs) accordingly in HIAs? How do environmental exposures (e.g., air pollution, temperature extremes, greenspace, noise) interact with physical activity behaviours and their health effects? How do activity timing, intensity, and location modify the interaction between physical activity and environmental risks?

## Data Availability

No data was used for the research described in the article.

## References

[R1] Aasvang Gunn Marit, Stockfelt Leo, Sørensen Mette, , 2023. Burden of disease due to transportation noise in the nordic countries. Environ. Res 231 (August), 116077. 10.1016/j.envres.2023.116077. 37156356

[R2] Abdulmajeed Jazeel, Furuya-Kanamori Luis, Chivese Tawanda, Xu Chang, Thalib Lukman, Suhail AR, 2025. Defining the exit meta-analysis. JBI Evidence Synthesis 23 (3), 480–492. 10.11124/JBIES-24-00155. 39252559

[R3] Agier Lydiane, Portengen Lützen, Chadeau-Hyam Marc, , 2016. A systematic comparison of linear regression–based statistical methods to assess exposome-health associations. Environ. Health Perspect 124 (12), 1848–1856. 10.1289/EHP172. 27219331 PMC5132632

[R4] Allen Craig D., Macalady Alison K., Chenchouni Haroun, , 2010. A global overview of drought and heat-induced tree mortality reveals emerging climate change risks for forests. Forest Ecology and Management, Adaptation of Forests and Forest Management to Changing Climate 259 (4), 660–684. 10.1016/j.foreco.2009.09.001.

[R5] Armstrong Ben, Bell Michelle L., Micheline de Sousa Zanotti Stagliorio Coelho, , 2017. Longer-term impact of high and low temperature on mortality: an international study to clarify length of mortality displacement. Environ. Health Perspect 125 (10), 107009. 10.1289/EHP1756. 29084393 PMC5933302

[R6] Aromataris Edoardo, Fernandez Ritin, Godfrey Christina M., Holly Cheryl, Khalil Hanan, Tungpunkom Patraporn, 2015. Summarizing systematic reviews: methodological development, conduct and reporting of an umbrella review approach. JBI Evidence Implementation 13 (3), 3. 10.1097/XEB.0000000000000055. 26360830

[R7] Asaad Safar M., Maghdid Halgurd S., 2022. A comprehensive review of indoor/outdoor localization solutions in IoT era: research challenges and future perspectives. Comput. Netw 212 (July), 109041. 10.1016/j.comnet.2022.109041.

[R8] Azzini Ivano, Listorti Giulia, Alex Mara Thierry, Rosati Rossana, 2020. Uncertainty and Sensitivity Analysis for Policy Decision Making: an Introductory Guide. Publications Office of the European Union. https://data.europa.eu/doi/10.2760/922129.

[R9] Barboza Evelise Pereira, Cirach Marta, Khomenko Sasha, , 2021. Green space and mortality in European cities: a health impact assessment study. Lancet Planet. Health 5 (10), 10. 10.1016/S2542-5196(21)00229-1. 34627476

[R10] Beenackers Marielle A., Kamphuis Carlijn BM., Giskes Katrina, , 2012. Socioeconomic inequalities in occupational, Leisure-time, and transport related physical activity among European adults: a systematic review. Int. J. Behav. Nutr. Phys. Activ 9 (1), 116. 10.1186/1479-5868-9-116. PMC3491027 22992350

[R11] Bell Sarah L., Wheeler Benedict W., Phoenix Cassandra, 2017. Using geonarratives to explore the diverse temporalities of therapeutic landscapes: perspectives from “Green” and “Blue” settings. Annals of the American Association of Geographers 107 (1), 93–108. 10.1080/24694452.2016.1218269.

[R12] Benavides Jaime, Rowland Sebastian T., Shearston Jenni A., Nunez Yanelli, Jack Darby W., Kioumourtzoglou Marianthi-Anna, 2022. Methods for evaluating environmental health impacts at different stages of the policy process in cities. Curr. Environ. Health Rep 9 (2), 183–195. 10.1007/s40572-022-00349-5. 35389203 PMC8986968

[R13] Benmarhnia T, Deguen S, Kaufman JS, Smargiassi A, 2015. Vulnerability to heat-related mortality: a systematic review, meta-analysis, and meta-regression analysis. Epidemiology 26 (6), 6. 10.1097/EDE.0000000000000375 rayyan-714269924. 26332052

[R14] Bernard Paquito, Chevance Guillaume, Kingsbury Celia, , 2021. Climate change, physical activity and sport: a systematic review. Sports Med. 51 (5), 1041–1059. 10.1007/s40279-021-01439-4. 33689139

[R15] Berry Helen Louise, Bowen Kathryn, Kjellstrom Tord, 2010. Climate change and mental health: a causal pathways framework. Int. J. Publ. Health 55 (2), 123–132. 10.1007/s00038-009-0112-0. 20033251

[R16] Birenboim Amit, Helbich Marco, Kwan Mei-Po, 2021. Advances in portable sensing for urban environments: understanding cities from a mobility perspective. Comput. Environ. Urban Syst 88 (July), 101650. 10.1016/j.compenvurbsys.2021.101650.

[R17] de Bont Jeroen, Pickford Regina, Åström Christopher, , 2023. Mixtures of long-term exposure to ambient air pollution, built environment and temperature and stroke incidence across Europe. Environ. Int 179 (September), 108136. 10.1016/j.envint.2023.108136. 37598594

[R18] Boogaard Hanna, Walker Katherine, Cohen Aaron J., 2019. Air pollution: the emergence of a major global health risk factor. International Health 11 (6), 417–421. 10.1093/inthealth/ihz078. 31613318

[R19] Boogaard Hanna, Atkinson Richard W., Brook Jeffrey R., , 2023. Evidence synthesis of observational studies in environmental health: lessons learned from a systematic review on traffic-related air pollution. Environ. Health Perspect 131 (11), 11. 10.1289/EHP11532. PMC10664749 37991444

[R20] Boogaard Hanna, Crouse Dan L., Tanner Eva, , 2024. Assessing adverse health effects of long-term exposure to low levels of ambient air pollution: the HEI experience and what’s next? Environmental Science & Technology. 10.1021/acs.est.3c09745 ahead of print, July 11. PMC11270999 38991107

[R21] Brauer Michael, Roth Gregory A., Aravkin Aleksandr Y., , 2024. Global burden and strength of evidence for 88 risk factors in 204 countries and 811 subnational locations, 1990–2021: a systematic analysis for the global burden of disease study 2021. Lancet 403 (10440), 2162–2203. 10.1016/S0140-6736(24)00933-4. 38762324 PMC11120204

[R22] Bressane Adriano, Pinto Joao Pedro da Cunha, Garcia Goulart Ana Paula, Líliam César de Castro Medeiros, 2024. Which dimensions of nature contact in urban green spaces Most significantly contribute to mental wellbeing? A multidimensional analysis in Brazilian metropolitan cities. Health Place 89 (September), 103335. 10.1016/j.healthplace.2024.103335. 39154413

[R23] Briggs David J., Sabel Clive E., Lee Kayoung, 2009. Uncertainty in epidemiology and health risk and impact assessment. Environ. Geochem. Health 31 (2), 189–203. 10.1007/s10653-008-9214-5. 18972068

[R24] Browning MHEM, Rigolon A, McAnirlin O, Yoon HV, 2022. Where greenspace matters Most: a systematic review of urbanicity, greenspace, and physical health. In: Landscape and Urban Planning (Department of Parks, Recreation and Tourism Management, Clemson University, 263 Lehotsky Hall, Clemson, SC, United States Department of City & Metropolitan Planning, the University of Utah, Salt Lake City, UT, United States Department of Parks, Recreation and Tourism Management, 217. Clemson University, United States Department of Recreation, Sport, and Tourism University of Illinois at Urbana-Champaign, Champaign, IL, United States. 10.1016/j.landurbplan.2021.104233.

[R25] Browning MHEM, Locke DH, Konijnendijk C, , 2024. Measuring the 3-30-300 rule to help cities meet nature access thresholds. Sci. Total Environ 907 (January), 167739. 10.1016/j.scitotenv.2023.167739. 37832672 PMC11090249

[R26] Brum Rodrigo de Lima, Penteado Júlia Oliveira, Ramires Paula Florencio, , 2024. Southern air project - Scientific efforts to monitor and measure the impacts of air pollution in Southern Brazil. Societal Impacts 4 (December), 100074. 10.1016/j.socimp.2024.100074.

[R27] Brunekreef Bert, Strak Maciej, Chen Jie, , 2021. Mortality and morbidity effects of long-term exposure to low-level PM2.5, BC, NO2, and O3: an analysis of European cohorts in the ELAPSE project. Research Reports. Health Effects Institute, p. 208, 2021 (September. PMC9476567 36106702

[R28] Brunekreef Bert, Straif Kurt, Pearce Neil, 2024. Reviewing umbrella reviews of systematic reviews of original studies on the effects of air pollution on disease. Environmental Epidemiology 8 (4), 4. 10.1097/EE9.0000000000000324. PMC11305730 39114736

[R29] Burkart Katrin G., Brauer Michael, Aravkin Aleksandr Y., , 2021. Estimating the cause-specific relative risks of non-optimal temperature on daily mortality: a two-part modelling approach applied to the global burden of disease study. Lancet 398 (10301), 685–697. 10.1016/S0140-6736(21)01700-1. 34419204 PMC8387975

[R30] Cai Jiannan, Kwan Mei-Po, 2024. The universal neighborhood effect averaging in mobility-dependent environmental exposures. Environmental Science & Technology 58 (45), 20030–20039. 10.1021/acs.est.4c02464. 39360926 PMC11562727

[R31] Castro Alberto, Röösli Martin, de Hoogh Kees, , 2022. Methods matter: a comparative review of health risk assessments for ambient air pollution in Switzerland. Public Health Rev. 43, 1604431. 10.3389/phrs.2022.1604431. 35465140 PMC9020261

[R32] Cerin Ester, Sallis James F., Salvo Deborah, , 2022. Determining thresholds for spatial urban design and transport features that support walking to create healthy and sustainable cities: findings from the IPEN adult study. Lancet Global Health 10 (6), e895–e906. 10.1016/S2214-109X(22)00068-7. 35561724 PMC9731787

[R33] Chadeau-Hyam Marc, Campanella Gianluca, Jombart Thibaut, , 2013. Deciphering the complex: methodological overview of statistical models to derive OMICS-based biomarkers. Environ. Mol. Mutagen 54 (7), 542–557. 10.1002/em.21797. 23918146

[R34] Chang Jer-Hwa, Lee Yueh-Lun, Chang Li-Te, , 2023. Climate change, air quality, and respiratory health: a focus on particle deposition in the lungs. Ann. Med 55 (2), 2. 10.1080/07853890.2023.2264881. PMC10561567 37801626

[R35] Charani Esmita, Abimbola Seye, Pai Madhukar, , 2022. Funders: the missing link in equitable global health research? PLOS Global Public Health 2 (6), e0000583. 10.1371/journal.pgph.0000583. 36962429 PMC10021882

[R36] Chastin Sebastien F.M., De Craemer Marieke, De Cocker Katrien, , 2019. How does light-intensity physical activity associate with adult cardiometabolic health and mortality? Systematic review with meta-analysis of experimental and observational studies. Br. J. Sports Med 53 (6), 6. rayyan–714270392. 10.1136/bjsports-2017-097563. 29695511 PMC6579499

[R37] Chen Jie, Hoek Gerard, 2020. Long-term exposure to PM and all-cause and cause-specific mortality: a systematic review and meta-analysis. Environ. Int 143 (October), 105974. 10.1016/j.envint.2020.105974. 32703584

[R38] Chen Jie, Braun Danielle, Christidis Tanya, , 2023. Long-term exposure to low-level PM2.5 and mortality: investigation of heterogeneity by harmonizing analyses in large cohort studies in Canada, United States, and Europe. Environ. Health Perspect 131 (12), 12. 10.1289/EHP12141. PMC10691665 38039140

[R39] Chen Xia, Liu Mingliang, Zuo Lei, , 2023. Environmental noise exposure and health outcomes: an umbrella review of systematic reviews and meta-analysis. Eur. J. Publ. Health 33 (4), 4. 10.1093/eurpub/ckad044. PMC11314258 37030015

[R40] Chen Yingxin, Hansell Anna L., Clark Sierra N., Cai Yutong Samuel, 2023. Environmental noise and health in low-middle-income-countries: a systematic review of epidemiological evidence. Environmental Pollution 316 (January), 120605. 10.1016/j.envpol.2022.120605. 36347406

[R41] Chen Jie, Hart Jaime E., Fisher Naomi D.L., , 2024. Multiple environmental exposures and the development of hypertension in a prospective US-Based cohort of female nurses: a mixture analysis. Environmental Science & Technology 58 (32), 14146–14157. 10.1021/acs.est.4c03722. 39083359 PMC12330077

[R42] Chen Kai, de Schrijver Evan, Sivaraj Sidharth, , 2024. Impact of population aging on future temperature-related mortality at different global warming levels. Nat. Commun 15 (1), 1796. 10.1038/s41467-024-45901-z. 38413648 PMC10899213

[R43] Chen Sujuan, Liu Di, Huang Lin, , 2024. Global associations between long-term exposure to PM2.5 constituents and health: a systematic review and meta-analysis of cohort studies. J. Hazard Mater 474 (August), 134715. 10.1016/j.jhazmat.2024.134715. 38838524

[R44] Chen Xuan, Gehring Ulrike, Dyer Georgia M.C., , 2024. Single- and two-pollutant concentration-response functions for PM2.5 and NO2 for quantifying mortality burden in health impact assessments. Environ. Res 263 (December), 120215. 10.1016/j.envres.2024.120215. 39448006

[R45] Chen Xuan, Gehring Ulrike, Dyer Georgia M.C., , 2025. Exposure-response functions of the correlated environmental exposures green space, noise, and air pollution for quantifying mortality burden in health impact assessment. Environ. Int 202 (August), 109645. 10.1016/j.envint.2025.109645. 40582334

[R46] Cheng Jian, Xu Zhiwei, Bambrick Hilary, Su Hong, Tong Shilu, Hu Wenbiao, 2019. Impacts of exposure to ambient temperature on burden of disease: a systematic review of epidemiological evidence. Int. J. Biometeorol 63 (8), 8. 10.1007/s00484-019-01716-y rayyan-714270485. 31011886

[R47] Choi Hayon Michelle, Lee Whanhee, Roye Dominic, , 2022. Effect modification of greenness on the association between heat and mortality: a multi-city multi-country study. EBioMedicine 84 (October), 104251. 10.1016/j.ebiom.2022.104251. 36088684 PMC9471476

[R48] Chowdhury Sourangsu, Dey Sagnik, 2016. Cause-specific premature death from ambient PM2.5 exposure in India: estimate adjusted for baseline mortality. Environ. Int 91 (May), 283–290. 10.1016/j.envint.2016.03.004. 27063285

[R49] Christidis Tanya, Pinault Lauren L., Crouse Dan L., Tjepkema Michael, 2021. The influence of outdoor PM2.5 concentration at workplace on nonaccidental mortality estimates in a Canadian census-based cohort. Environmental Epidemiology (Philadelphia, Pa.) 5 (6), e180. 10.1097/EE9.0000000000000180. 34909560 PMC8663884

[R50] Clark Sierra N., Alli Abosede S., Nathvani Ricky, , 2021. Space-time characterization of community noise and sound sources in Accra, Ghana. Sci. Rep 11 (1), 11113. 10.1038/s41598-021-90454-6. 34045545 PMC8160008

[R51] Clark Sierra N., Alli Abosede S., Ezzati Majid, , 2022. Spatial modelling and inequalities of environmental noise in Accra, Ghana. Environ. Res 214 (November), 113932. 10.1016/j.envres.2022.113932. 35868576 PMC9441709

[R52] COMEAP. (in preparation). ‘Statement on COMEAP’s Updated Views on the Shape of the Concentration-Response Curve Linking Long-Term Exposure to PM2.5 with All-Cause Mortality Risk’ Committee on the Medical Effects of Air Pollutants.

[R53] COMEAP, 2018. Associations of long-term average concentrations of nitrogen dioxide with mortality. Committee on the Medical Effects of Air Pollutants. https://www.gov.uk/government/publications/nitrogen-dioxide-effects-on-mortality.

[R54] COMEAP, 2020. ‘Statement on the Evidence for Health Effects Associated with Exposure to Non-Exhaust Particulate Matter from Road Transport.’. Committee on the Medical Effects of Air Pollutants. https://www.gov.uk/government/publications/non-exhaust-particulate-matter-from-road-transport-health-effects.

[R55] COMEAP, 2022a. Statement on quantifying mortality associated with long-term exposure to PM2.5 Committee on the Medical Effects of Air Pollutants. https://www.gov.uk/government/publications/particulate-air-pollution-quantifying-effects-on-mortality.

[R56] COMEAP, 2022b. Statement on the differential toxicity of particulate matter according to source or constituents. Committee on the Medical Effects of Air Pollutants. https://www.gov.uk/government/publications/particulate-air-pollution-health-effects-of-exposure.

[R57] COMEAP, 2025. Working Paper 1. Susceptibility of Population Groups to Air Pollution Committee on the Medical Effects of Air Pollutants. March 18. https://www.gov.uk/government/publications/advice-given-to-the-air-quality-information-system-aqis-review-steering-group/working-paper-1-susceptibility-of-population-groups-to-air-pollution.

[R58] Cooke Steven J., Cook Carly N., Nguyen Vivian M., , 2023. Environmental evidence in action: on the science and practice of evidence synthesis and evidence-based decision-making. Environ. Evid 12 (1), 10. 10.1186/s13750-023-00302-5. 37220478 PMC10191815

[R59] Cordiner Rhiannon, Wan Kai, Hajat Shakoor, Macintyre Helen L., 2024. Accounting for adaptation when projecting climate change impacts on health: a review of temperature-related health impacts. Environ. Int 188 (June), 108761. 10.1016/j.envint.2024.108761. 38788417

[R60] Cox Louis Anthony, 2020. Implications of nonlinearity, confounding, and interactions for estimating exposure concentration-response functions in quantitative risk analysis. Environ. Res 187 (August), 109638. 10.1016/j.envres.2020.109638. 32450424 PMC7235595

[R61] Crouse Dan L., Peters Paul A., Hystad Perry, , 2015. Ambient PM2.5, O_3_, and NO_2_ exposures and associations with mortality over 16 years of Follow-Up in the Canadian census health and environment cohort (CanCHEC). Environ. Health Perspect 123 (11), 1180–1186. 10.1289/ehp.1409276. 26528712 PMC4629747

[R62] Crouse Dan L., Erickson Anders C., Christidis Tanya, , 2020. Evaluating the sensitivity of PM2.5-Mortality associations to the spatial and temporal scale of exposure assessment. Epidemiology 31 (2), 168–176. 10.1097/EDE.0000000000001136. 31693516

[R63] Cumpston M, Flemyng E, 2024. Chapter IV: updating a review. In: Cochrane Handbook for Systematic Reviews of Interventions, 6.5 Cochrane.

[R64] van Daalen Kim R, Tonne Cathryn, Semenza Jan C., , 2024. The 2024 Europe report of the lancet countdown on health and climate change: unprecedented warming demands unprecedented action. Lancet Public Health 9 (7), e495–e522. 10.1016/S2468-2667(24)00055-0. 38749451 PMC11209670

[R65] Dean Julie H., Shanahan Danielle F., Bush Robert, , 2018. Is nature relatedness associated with better mental and physical health? Int. J. Environ. Res. Publ. Health 15 (7), 7. 10.3390/ijerph15071371. PMC6069224 29966307

[R66] Deivanayagam Thilagawathi Abi, English Sonora, Hickel Jason, , 2023. Envisioning environmental equity: climate change, health, and racial justice. Lancet 402 (10395), 64–78. 10.1016/S0140-6736(23)00919-4. 37263280 PMC10415673

[R67] Dempsey Paddy C., Rowlands Alex V., Strain Tessa, , 2022. Physical activity volume, intensity, and incident cardiovascular disease. Eur. Heart J 43 (46), 4789–4800. 10.1093/eurheartj/ehac613. 36302445

[R68] deSouza Priyanka N., Anenberg Susan, Neal Fann, , 2024. Evaluating the sensitivity of mortality attributable to pollution to modeling choices: a case study for Colorado. Environ. Int 185 (March), 108416. 10.1016/j.envint.2024.108416. 38394913

[R69] Dimakopoulou Konstantina, Nobile Federica, de Bont Jeroen, , 2024. Disentangling associations between multiple environmental exposures and all-cause mortality: an analysis of European administrative and traditional cohorts. Frontiers in Epidemiology 3 (January), 1328188. 10.3389/fepid.2023.1328188. 38455945 PMC10910955

[R70] Ding Ding, Ekelund Ulf, 2024. From London buses to activity trackers: a reflection of 70 years of physical activity research. Journal of Sport and Health Science 13 (6), 736–738. 10.1016/j.jshs.2024.06.001. 38851584 PMC11336341

[R71] Doherty Brett T., Koelmel Jeremy P., Lin Elizabeth Z., Romano Megan E., Godri Pollitt Krystal J., 2021. Use of exposomic methods incorporating sensors in environmental epidemiology. Curr. Environ. Health Rep 8 (1), 34–41. 10.1007/s40572-021-00306-8. 33569731

[R72] Dominici Francesca, Peng Roger D., Barr Christopher D., Bell Michelle L., 2010. Protecting human health from air pollution: shifting from a single-pollutant to a multi-pollutant approach. Epidemiology 21 (2), 187–194. 10.1097/EDE.0b013e3181cc86e8. 20160561 PMC3478072

[R73] Dong Jiaying, Browning Matthew H.E. M., Reuben Aaron, , 2024. The paradox of high greenness and poor health in rural central appalachia. Environ. Res 248 (May), 118400. 10.1016/j.envres.2024.118400. 38309568 PMC11253236

[R74] van Donkelaar Aaron, Martin Randall V., Li Chi, Burnett Richard T., 2019. Regional estimates of chemical composition of fine particulate matter using a combined geoscience-statistical method with information from satellites, models, and monitors. Environmental Science & Technology 53 (5), 2595–2611. 10.1021/acs.est.8b06392. 30698001

[R75] Donovan Geoffrey H., Gatziolis Demetrios, Derrien Monika, Michael Yvonne L., Prestemon Jeffrey P., Douwes Jeroen, 2022. Shortcomings of the normalized difference vegetation index as an exposure metric. Nat. Plants 8 (6), 617–622. 10.1038/s41477-022-01170-6. 35697731

[R76] Dyer Georgia M.C., Khomenko Sasha, Adlakha Deepti, Anenberg Susan, Angelova Julianna, , 2024a. Commentary: a road map for future data-driven urban planning and environmental health research. Cities 155 (December), 105340. 10.1016/j.cities.2024.105340. 39351125 PMC7616649

[R77] Dyer Georgia M.C., Khomenko Sasha, Adlakha Deepti, Anenberg Susan, Behnisch Martin, , 2024b. Exploring the nexus of urban form, transport, environment and health in large-scale urban studies: a state-of-the-art scoping review. Environ. Res 257 (September), 119324. 10.1016/j.envres.2024.119324. 38844028 PMC7617738

[R78] Dzhambov Angel, Hartig Terry, Markevych Iana, Tilov Boris, Dimitrova Donka, 2018. Urban residential greenspace and mental health in youth: different approaches to testing multiple pathways yield different conclusions. Environ. Res 160 (January), 47–59. 10.1016/j.envres.2017.09.015. 28961469

[R79] Dzhambov Angel M., Browning Matthew H.E. M., Markevych Iana, Hartig Terry, Lercher Peter, 2020. Analytical approaches to testing pathways linking greenspace to health: a scoping review of the empirical literature. Environ. Res 186 (July), 109613. 10.1016/j.envres.2020.109613. 32668553

[R80] EEA, 2022. Health Impacts of Air Pollution in Europe, 2022’. Briefing. European Environment Agency. https://www.eea.europa.eu/publications/air-quality-in-europe-2022/health-impacts-of-air-pollution.

[R81] EEA, 2025. ‘Environmental noise in Europe 2025’. June 23. https://www.eea.europa.eu/en/analysis/publications/environmental-noise-in-europe-2025.

[R82] Eick Stephanie M., Goin Dana E., Chartres Nicholas, Lam Juleen, Woodruff Tracey J., 2020. Assessing risk of bias in human environmental epidemiology studies using three tools: different conclusions from different tools. Syst. Rev 9 (1), 249. 10.1186/s13643-020-01490-8. 33121530 PMC7596989

[R83] Eisen Aaron M., Bratman Gregory N., Olvera-Alvarez Hector A., 2024. Susceptibility to stress and nature exposure: unveiling differential susceptibility to physical environments; a randomized controlled trial. PLoS One 19 (4), e0301473. 10.1371/journal.pone.0301473. 38630650 PMC11023441

[R84] Ekelund Ulf, Tarp Jakob, Steene-Johannessen Jostein, , 2019. Dose-response associations between accelerometry measured physical activity and sedentary time and all cause mortality: systematic review and harmonised meta-analysis. Br. Med. J 366, l4570. 10.1136/bmj.l4570 rayyan-714271105. 31434697 PMC6699591

[R85] Ekelund Ulf, Sanchez-Lastra Miguel Adriano, Dalene Knut Eirik, Tarp Jakob, 2024a. Dose-response associations, physical activity intensity and mortality risk: a narrative review. Journal of Sport and Health Science 13 (1), 24–29. 10.1016/j.jshs.2023.09.006. 37734548 PMC10818107

[R86] Ekelund Ulf, Tarp Jakob, Sanchez-Lastra Miguel Adriano, Dalene Knut Erik, 2024b. Physical activity, sedentary time and health - a narrative review with new insights. Danish Medical Journal 71 (11), A06240433. 10.61409/A06240433. 39575942

[R87] Elliott Julian H., Synnot Anneliese, Turner Tari, , 2017. Living systematic review: 1. Introduction-the why, what, when, and how. J. Clin. Epidemiol 91 (November), 23–30. 10.1016/j.jclinepi.2017.08.010. 28912002

[R88] Eminson K, Cai YS, Chen Y, , 2023. Does air pollution confound associations between environmental noise and cardiovascular outcomes? - a systematic review. Environ. Res 232. 10.1016/j.envres.2023.116075. 37182833

[R89] Engelmann Nicole, Blanes Guàrdia Núria, Fons-Esteve Jaume, Vienneau Danielle, Peris Eulália, Röösli Martin, 2023. Environmental noise health risk assessment: methodology for assessing health risks using data reported under the environmental noise directive. Eionet Portal. https://www.eionet.europa.eu/etcs/etc-he/products/etc-he-products/etc-he-reports/etc-he-report-2023-11-environmental-noise-health-risk-assessment-methodology-for-assessing-health-risks-using-data-reported-under-the-environmental-noise-directive.

[R90] Europe, The Lancet Regional Health-, 2023. Noise pollution: more attention is needed. The Lancet Regional Health – Europe 24 (January). 10.1016/j.lanepe.2022.100577. PMC9832265 36643665

[R91] Evangelopoulos Dimitris, Katsouyanni Klea, Schwartz Joel, Walton Heather, 2021. Quantifying the short-term effects of air pollution on health in the presence of exposure measurement error: a simulation study of multi-pollutant model results. Environmental Health 20 (1), 94. 10.1186/s12940-021-00757-4. 34429109 PMC8385952

[R92] Fann Neal, Bell Michelle L., Walker Katy, Bryan Hubbell, 2011. Improving the linkages between air pollution epidemiology and quantitative risk assessment. Environ. Health Perspect 119 (12), 12. 10.1289/ehp.1103780. PMC3261990 21816702

[R93] Fenech Sara, Doherty Ruth M., Heaviside Clare, Vardoulakis Sotiris, Macintyre Helen L., O’Connor Fiona M., 2018. The influence of model spatial resolution on simulated ozone and fine particulate matter for Europe: implications for health impact assessments. Atmos. Chem. Phys 18 (8), 5765–5784. 10.5194/acp-18-5765-2018.

[R94] Ferguson Lauren, Taylor Jonathon, Zhou Ke, , 2021. Systemic inequalities in indoor air pollution exposure in London, UK. Buildings & Cities 2 (1). 10.5334/bc.100. PMC7610964 34124667

[R95] Ferguson Lauren, Taylor Jonathon, Symonds Phil, Davies Michael, Dimitroulopoulou Sani, 2023. Analysis of inequalities in personal exposure to PM2.5: a modelling study for the greater London school-aged population. Sci. Total Environ 905 (December), 167056. 10.1016/j.scitotenv.2023.167056. 37717780

[R96] Folkerts Mireille A., Bröde Peter, Wouter Botzen WJ, , 2022. Sex differences in temperature-related all-cause mortality in the Netherlands. Int. Arch. Occup. Environ. Health 95 (1), 249–258. 10.1007/s00420-021-01721-y. 34089351 PMC8755659

[R97] Foraster Maria, Künzli Nino, Aguilera Inmaculada, , 2014. High blood pressure and long-term exposure to indoor noise and air pollution from road traffic. Environ. Health Perspect 122 (11), 1193–1200. 10.1289/ehp.1307156. 25003348 PMC4216159

[R98] Forastiere Francesco, Orru Hans, Krzyzanowski Michal, Spadaro Joseph V., 2024a. The last decade of air pollution epidemiology and the challenges of quantitative risk assessment. Environmental Health 23 (1), 98. 10.1186/s12940-024-01136-5. 39543692 PMC11566658

[R99] Forastiere Francesco, Spadaro Joseph V., Ancona Carla, , 2024b. Choices of morbidity outcomes and concentration–response functions for health risk assessment of long-term exposure to air pollution. Environmental Epidemiology 8 (4), 4. 10.1097/EE9.0000000000000314. PMC11265782 39045486

[R100] Garber Michael D., Benmarhnia Tarik, de Nazelle Audrey, Nieuwenhuijsen Mark, Rojas-Rueda David, 2024. The Epidemiologic Case for Urban Health: Conceptualizing and Measuring the Magnitude of Challenges and Potential Benefits, 13. F1000Research, p. 950. 10.12688/f1000research.154967.1. August 22. 40110549 PMC11920689

[R101] Garcia Leandro, Pearce Matthew, Ali Abbas, , 2023. Non-occupational physical activity and risk of cardiovascular disease, cancer and mortality outcomes: a dose-response meta-analysis of large prospective studies. Br. J. Sports Med 57 (15), 15. 10.1136/bjsports-2022-105669 rayyan-714271462. PMC10423495 36854652

[R102] García-León David, Masselot Pierre, Mistry Malcolm N., , 2024. Temperature-related mortality burden and projected change in 1368 European regions: a modelling study. Lancet Public Health 9 (9), e644–e653. 10.1016/S2468-2667(24)00179-8. 39181156

[R103] Gasparrini Antonio, Guo Yuming, Hashizume Masahiro, , 2015. Mortality risk attributable to high and low ambient temperature: a multicountry observational study. Lancet (London, England) 386 (9991), 369–375. 10.1016/S0140-6736(14)62114-0. 26003380 PMC4521077

[R104] Gasparrini Antonio, Masselot Pierre, Scortichini Matteo, , 2022. Small-area assessment of temperature-related mortality risks in England and Wales: a case time series analysis. Lancet Planet. Health 6 (7), e557–e564. 10.1016/S2542-5196(22)00138-3. 35809585

[R105] Gasparrini Antonio, Vicedo-Cabrera Ana Maria, Tobias Aurelio, 2024. The multi-country multi-city collaborative research network: an international research consortium investigating environment, climate, and health. Environmental Epidemiology 8 (5), e339. 10.1097/EE9.0000000000000339. 39263673 PMC11390054

[R106] Gates LS, Leyland KM, Sheard S, , 2017. Physical activity and osteoarthritis: a consensus study to harmonise self-reporting methods of physical activity across international cohorts. Rheumatol. Int 37 (4), 469–478. 10.1007/s00296-017-3672-y. 28238075 PMC5357277

[R107] Giallouros Giorgos, Kouis Panayiotis, Papatheodorou Stefania I., Woodcock James, Tainio Marko, 2020. The long-term impact of restricting cycling and walking during high air pollution days on all-cause mortality: health impact assessment study. Environ. Int 140 (July), 105679. 10.1016/j.envint.2020.105679. 32353667

[R108] Glazener Andrew, Sanchez Kristen, Ramani Tara, , 2021. Fourteen pathways between urban transportation and health: a conceptual model and literature review. J. Transport Health 21 (June), 101070. 10.1016/j.jth.2021.101070.

[R109] Gowers Alison M., Walton Heather, Exley Karen S., Fintan Hurley J, 2020. Using epidemiology to estimate the impact and burden of exposure to air pollutants. Philosophical Transactions. Series A, Mathematical, Physical, and Engineering Sciences 378 (2183), 20190321. 10.1098/rsta.2019.0321. 32981441 PMC7536035

[R110] Green Hunter, Bailey Jennifer, Schwarz Lara, Vanos Jennifer, Ebi Kristie, Benmarhnia Tarik, 2019. Impact of heat on mortality and morbidity in low and middle income countries: a review of the epidemiological evidence and considerations for future research. Environ. Res 171 (April), 80–91. 10.1016/j.envres.2019.01.010. 30660921

[R111] Guo Yuming, Gasparrini Antonio, Armstrong Ben, , 2014. Global variation in the effects of ambient temperature on mortality: a systematic evaluation. Epidemiology 25 (6), 781–789. 10.1097/EDE.0000000000000165. 25166878 PMC4180721

[R112] Hahad Omar, Daiber Andreas, Münzel Thomas, 2023. Physical activity in polluted air: an urgent call to study the health risks. Lancet Planet. Health 7 (4), e266–e267. 10.1016/S2542-5196(23)00055-4. 37019566

[R113] Hajat Anjum, MacLehose Richard F., Rosofsky Anna, Walker Katherine D., Clougherty Jane E., 2021. Confounding by socioeconomic status in epidemiological studies of air pollution and health: challenges and opportunities. Environ. Health Perspect 129 (6), 65001. 10.1289/EHP7980. 34124937 PMC8202292

[R114] Hanna Edward, Francis Jennifer, Wang Muyin, , 2024. Influence of high-latitude blocking and the northern stratospheric polar vortex on cold-air outbreaks under arctic amplification of global warming. Environ. Res.: Climate 3 (4), 042004. 10.1088/2752-5295/ad93f3.

[R115] Hao Guang, Zuo Lei, Weng Xueqiong, , 2022. Associations of road traffic noise with cardiovascular diseases and mortality: longitudinal results from UK biobank and meta-analysis. Environ. Res 212 (September), 113129. 10.1016/j.envres.2022.113129. 35358546

[R116] Hao Hua, Wang Yifan, Zhu Qiao, , 2023. National cohort study of long-term exposure to PM2.5 components and mortality in medicare American older adults. Environmental Science & Technology 57 (17), 6835–6843. 10.1021/acs.est.2c07064. 37074132 PMC10157884

[R117] Health Effects Institute, 2022. Systematic Review and Meta-Analysis of Selected Health Effects of Long-Term Exposure to Traffic-Related Air Pollution. Health Effects Institute. June 16. https://www.healtheffects.org/publication/systematic-review-and-meta-analysis-selected-health-effects-long-term-exposure-traffic.

[R118] Helbig Carolin, Ueberham Maximilian, Becker Anna Maria, Marquart Heike, Schlink Uwe, 2021. Wearable sensors for human environmental exposure in urban settings. Curr. Pollut. Rep 7 (3), 417–433. 10.1007/s40726-021-00186-4.

[R119] Héritier Harris, Vienneau Danielle, Foraster Maria, , 2019. A systematic analysis of mutual effects of transportation noise and air pollution exposure on myocardial infarction mortality: a nationwide cohort study in Switzerland. Eur. Heart J 40 (7), 598–603. 10.1093/eurheartj/ehy650. 30357335

[R120] Higgins Julian P.T., Morgan Rebecca L., Rooney Andrew A., , 2024. A tool to assess risk of bias in non-randomized Follow-up studies of exposure effects (ROBINS-E). Environ. Int 186 (April), 108602. 10.1016/j.envint.2024.108602. 38555664 PMC11098530

[R121] Hoek Gerard, Krishnan Ranjini M., Beelen Rob, , 2013. Long-term air pollution exposure and Cardio- respiratory mortality: a review. Environmental Health 12 (1), 43. 10.1186/1476-069X-12-43. 23714370 PMC3679821

[R122] Hoek Gerard, Vienneau Danielle, de Hoogh Kees, 2024. Does residential address-based exposure assessment for outdoor air pollution lead to bias in epidemiological studies? Environmental Health: A Global Access Science Source 23 (1), 75. 10.1186/s12940-024-01111-0. 39289774 PMC11406750

[R123] Holland Isabel, DeVille Nicole V., Browning Matthew H.E. M., , 2021. Measuring nature contact: a narrative review. Int. J. Environ. Res. Publ. Health 18 (8), 4092. 10.3390/ijerph18084092. PMC8069863 33924490

[R124] Holtermann Andreas, Krause Niklas, van der Beek Allard J., Straker Leon, 2018. The physical activity paradox: six reasons why occupational physical activity (OPA) does not confer the cardiovascular health benefits that leisure time physical activity does. Br. J. Sports Med 52 (3), 149–150. 10.1136/bjsports-2017-097965. 28798040

[R125] Hou Yuchen, Cao Bin, Zhu Yingxin, , 2023. Temporal and spatial heterogeneity of indoor and outdoor temperatures and their relationship with thermal sensation from a global perspective. Environ. Int 179 (September), 108174. 10.1016/j.envint.2023.108174. 37660634

[R126] Hoyle Helen, Jorgensen Anna, Hitchmough James D., 2019. What determines how we see nature? Perceptions of naturalness in designed urban green spaces. People and Nature 1 (2), 167–180. 10.1002/pan3.19.

[R127] Huangfu Peijue, Atkinson Richard, 2020. Long-term exposure to NO2 and O3 and all-cause and respiratory mortality: a systematic review and meta-analysis. Environ. Int 144 (November), 105998. 10.1016/j.envint.2020.105998. 33032072 PMC7549128

[R128] Hwang Yun Hye, Roscoe Charlotte J., 2017. Preference for site conservation in relation to On-Site biodiversity and perceived site attributes: an on-Site survey of unmanaged urban greenery in a tropical city. Urban For. Urban Green 28 (December), 12–20. 10.1016/j.ufug.2017.09.011.

[R129] IHME, 2024. Epi Visualization. Institute for Health Metrics and Evaluation, University of Washington, Version Seattle, WA, 2024. Available. http://vizhub.healthdata.org/epi.

[R130] Ioannidis John P.A., 2016. The mass production of redundant, misleading, and conflicted systematic reviews and meta-analyses. Milbank Q. 94 (3), 485–514. 10.1111/1468-0009.12210. 27620683 PMC5020151

[R131] IPCC, 2023. ‘Climate Change 2023: Synthesis Report. Contribution of Working Groups I, II and III to the Sixth Assessment Report of the Intergovernmental Panel on Climate Change’. IPCC, Geneva, Switzerland.

[R132] Iungman Tamara, Cirach Marta, Marando Federica, , 2023. Cooling cities through urban green infrastructure: a health impact assessment of European cities. Lancet 401 (10376), 10376. 10.1016/S0140-6736(22)02585-5. 36736334

[R133] Iungman Tamara, Ventura Caballé Sergi, Segura-Barrero Ricard, , 2025. Co-Benefits of nature-based solutions: a health impact assessment of the Barcelona green corridor (*Eixos Verds*) plan. Environ. Int 196 (February), 109313. 10.1016/j.envint.2025.109313. 39919507 PMC11839897

[R134] Jaikumar R, Saab GB, Khreis H, Venugopal M, Ramani T, Kamal A, Davidson K, Bailey C, Deshmukh P, Baldauf R, 2025. Modeling traffic-related air pollution burden of disease using high spatial resolution data. J. Transport Health 44, 102155. 10.1016/j.jth.2025.102155 PMC12973170 41816774

[R135] Jephcote Calvin, Clark Sierra N., Hansell Anna L., , 2023. Spatial assessment of the attributable burden of disease due to transportation noise in England. Environ. Int 178 (August), 107966. 10.1016/j.envint.2023.107966. 37390771

[R136] Jerrett Michael, Donaire-Gonzalez David, Popoola Olalekan, , 2017. Validating novel air pollution sensors to improve exposure estimates for epidemiological analyses and citizen science. Environ. Res 158 (October), 286–294. 10.1016/j.envres.2017.04.023. 28667855

[R137] Jimenez Raquel B., Lane Kevin J., Hutyra Lucy R., Patricia Fabian M, 2022. Spatial resolution of normalized difference vegetation index and greenness exposure misclassification in an urban cohort. J. Expo. Sci. Environ. Epidemiol 32 (2), 2. 10.1038/s41370-022-00409-w. PMC11649244 35094014

[R138] Josey Kevin P., Delaney Scott W., Wu Xiao, , 2023. Air pollution and mortality at the intersection of race and social class. N. Engl. J. Med 388 (15), 15. 10.1056/NEJMsa2300523. PMC10182569 36961127

[R139] Kärmeniemi Mikko, Lankila Tiina, Ikäheimo Tiina, Koivumaa-Honkanen Heli, Korpelainen Raija, 2018. The built environment as a determinant of physical activity: a systematic review of longitudinal studies and natural experiments. Ann. Behav. Med 52 (3), 239–251. 10.1093/abm/kax043. 29538664

[R140] Kasdagli Maria-Iosifina, Orellano Pablo, Velasco Román Pérez, Samoli Evangelia, 2024. Long-term exposure to nitrogen dioxide and ozone and mortality: update of the WHO air quality guidelines systematic review and meta-analysis. Int. J. Publ. Health 69 (October), 1607676. 10.3389/ijph.2024.1607676. PMC11527649 39494092

[R141] Katsouyanni Klea, Evangelopoulos Dimitris, 2022. Invited perspective: impact of exposure measurement error on effect estimates—an important and neglected problem in air pollution epidemiology. Environ. Health Perspect 130 (7), 071302. 10.1289/EHP11277. 35904518 PMC9337231

[R142] Keil Alexander P., Buckley Jessie P., O’Brien Katie M., Ferguson Kelly K., Zhao Shanshan, White Alexandra J., 2020. A quantile-based g-Computation approach to addressing the effects of exposure mixtures. Environ. Health Perspect 128 (4), 047004. 10.1289/EHP5838. 32255670 PMC7228100

[R143] Kerr Jacqueline, Duncan Scott, Schipperjin Jasper, 2011. Using global positioning systems in health research: a practical approach to data collection and processing. Am. J. Prev. Med 41 (5), 532–540. 10.1016/j.amepre.2011.07.017. 22011426

[R144] Khomenko Sasha, Nieuwenhuijsen Mark, Ambròs Albert, Wegener Sandra, Mueller Natalie, 2020. Is a liveable city a healthy city? Health impacts of urban and transport planning in Vienna, Austria. Environ. Res 183 (April), 109238. 10.1016/j.envres.2020.109238. 32062485

[R145] Khomenko Sasha, Cirach Marta, Pereira-Barboza Evelise, , 2021. Premature mortality due to air pollution in European cities: a health impact assessment. Lancet Planet. Health 5 (3), 3. 10.1016/S2542-5196(20)30272-2. 33482109

[R146] Khomenko Sasha, Cirach Marta, Barrera-Gómez Jose, , 2022. Impact of road traffic noise on annoyance and preventable mortality in European cities: a health impact assessment. Environ. Int 162 (April), 107160. 10.1016/j.envint.2022.107160. 35231841

[R147] Khomenko Sasha, Burov Angel, Dzhambov Angel M., , 2025. Health burden and inequities of urban environmental stressors in Sofia, Bulgaria. Environ. Res 279 (August), 121782. 10.1016/j.envres.2025.121782. 40345423

[R148] Khreis Haneen, Johnson Jeremy, Jack Katherine, Dadashova Bahar, Park Eun Sug, 2022. Evaluating the performance of low-cost air quality monitors in Dallas, Texas. Int. J. Environ. Res. Publ. Health 19 (3), 3. 10.3390/ijerph19031647. PMC8835131 35162669

[R149] Khreis Haneen, Williams Harry, Abdollahpour Seyed Sajjad, , 2024. The nexus of transportation, the built environment, air pollution and health. Cities & Health 0 (0). 10.1080/23748834.2024.2376389.

[R150] Klompmaker Jochem O., Hoek Gerard, Bloemsma Lizan D., , 2018. Green space definition affects associations of green space with overweight and physical activity. Environ. Res 160 (January), 531–540. 10.1016/j.envres.2017.10.027. 29106952

[R151] Klompmaker Jochem O., Janssen Nicole A.H., Bloemsma Lizan D., , 2019. Associations of combined exposures to surrounding green, air pollution, and road traffic noise with cardiometabolic diseases. Environ. Health Perspect 127 (8), 087003. 10.1289/EHP3857. 31393793 PMC6792364

[R152] Klompmaker Jochem O., Hoek Gerard, Bloemsma Lizan D., , 2020. Surrounding green, air pollution, traffic noise exposure and non-accidental and cause-specific mortality. Environ. Int 134 (January), 105341. 10.1016/j.envint.2019.105341. 31783239

[R153] Klompmaker Jochem O., Janssen Nicole A.H., Bloemsma Lizan D., , 2021. Effects of exposure to surrounding green, air pollution and traffic noise with non-accidental and cause-specific mortality in the Dutch national cohort. Environmental Health: A Global Access Science Source 20 (1), 82. 10.1186/s12940-021-00769-0. 34261495 PMC8281461

[R154] Knobel Pablo, Maneja Roser, Bartoll Xavier, , 2021. Quality of urban green spaces influences residents’ use of these spaces, physical activity, and overweight/obesity. Environmental Pollution 271 (February), 116393. 10.1016/j.envpol.2020.116393. 33388678

[R155] Knol Anne B., Petersen Arthur C., van der Sluijs Jeroen P., Lebret Erik, 2009. Dealing with uncertainties in environmental burden of disease assessment. Environmental Health 8 (1), 21. 10.1186/1476-069X-8-21. 19400963 PMC2684742

[R156] Kondo Michelle C., Fluehr Jaime M., McKeon Thomas, Branas Charles C., 2018. Urban green space and its impact on human health. Int. J. Environ. Res. Publ. Health 15 (3), 445. 10.3390/ijerph15030445. PMC5876990 29510520

[R157] Kong Pei-Rou, Han Ke-Tsung, 2024. Psychological and physiological effects of soundscapes: a systematic review of 25 experiments in the English and Chinese literature. Sci. Total Environ 929 (June), 172197. 10.1016/j.scitotenv.2024.172197. 38582113

[R158] Korhonen Antti, Lehtomäki Heli, Rumrich Isabell, , 2019. Influence of spatial resolution on population PM2.5 exposure and health impacts. Air Qual. Atmos. Health 12 (6), 705–718. 10.1007/s11869-019-00690-z.

[R159] Kotsila Panagiota, Anguelovski Isabelle, 2023. Justice should be at the centre of assessments of climate change impacts on health. Lancet Public Health 8 (1), e11–e12. 10.1016/S2468-2667(22)00320-6. 36603904

[R160] Kragh Jørgen, Thysell Erik, Finne Per, Pedersen Frank, Michelsen Lene, Fryd Jakob, 2023. The Nord2000 prediction method for road traffic noise-outline and validation, and application in environmental noise mapping. J. Acoust. Soc. Am 154 (1), 547–555. 10.1121/10.0020275. 37504375

[R161] Kruize Hanneke, Van Kamp Irene, Van Den Berg Magdalena, , 2020. Exploring mechanisms underlying the relationship between the natural outdoor environment and health and well-being – results from the PHENOTYPE project. Environ. Int 134 (January), 105173. 10.1016/j.envint.2019.105173. 31677803

[R162] Ku Po-Wen, Hamer Mark, Liao Yung, Hsueh Ming-Chun, Chen Li-Jung, 2020. Device-measured light-intensity physical activity and mortality: a meta-analysis. Scandinavian Journal of Medicine & Science in Sports, Comment in: Scand. J. Med. Sci. Sports 30 (6), 1083–1084. 10.1111/sms.13557. https://www.ncbi.nlm.nih.gov/pubmed/32316073. 31545531

[R163] Kumar Ramya, Khosla Rajat, McCoy David, 2024. Decolonising global health research: shifting power for transformative change. PLOS Global Public Health 4 (4), e0003141. 10.1371/journal.pgph.0003141. 38656955 PMC11042701

[R164] Kwan Mei-Po, 2012. The uncertain geographic context problem. Ann. Assoc. Am. Geogr 102 (5), 958–968. 10.1080/00045608.2012.687349.

[R165] Kwan Mei-Po, 2018. The neighborhood effect averaging problem (NEAP): an elusive confounder of the neighborhood effect. Int. J. Environ. Res. Publ. Health 15 (9), 1841. 10.3390/ijerph15091841. PMC6163400 30150510

[R166] Labib SM, Huck Jonny J., Sarah Lindley, 2021. Modelling and mapping eye-level greenness visibility exposure using multi-source data at high spatial resolutions. Sci. Total Environ 755 (February), 143050. 10.1016/j.scitotenv.2020.143050. 33129523 PMC7562921

[R167] Lawlor Debbie A., Tilling Kate, George Davey Smith, 2016. Triangulation in aetiological epidemiology. Int. J. Epidemiol 45 (6), 1866–1886. 10.1093/ije/dyw314. 28108528 PMC5841843

[R168] Lee Whanhee, Choi Munjeong, Bell Michelle L., , 2022. Effects of urbanization on vulnerability to heat-related mortality in urban and rural areas in South Korea: a nationwide district-level time-series study. Int. J. Epidemiol 51 (1), 111–121. 10.1093/ije/dyab148. 34386817

[R169] Lee Duncan, Walton Heather, Evangelopoulos Dimitris, , 2023. Health impact assessment for air pollution in the presence of regional variation in effect sizes: the implications of using different meta-analytic approaches. Environmental Pollution 336 (November), 122465. 10.1016/j.envpol.2023.122465. 37640226

[R170] Lee Eun-Young, Park Seiyeong, Kim Yeong-Bae, , 2024. Exploring the interplay between climate change, 24-Hour movement behavior, and health: a systematic review. J. Phys. Activ. Health 21 (12), 1227–1245. 10.1123/jpah.2023-0637. 39187251

[R171] Lee Doo Hong, Chamberlain Brent, Park Hye Yeon, 2025. Toward a construct-based definition of urban green space: a literature review of the spatial dimensions of measurement, methods, and exposure. Land 14 (3), 3. 10.3390/land14030517.

[R172] Lesko Catherine R., Buchanan Ashley L., Westreich Daniel, Edwards Jessie K., Hudgens Michael G., Cole Stephen R., 2017. Generalizing study results: a potential outcomes perspective. Epidemiology 28 (4), 553. 10.1097/EDE.0000000000000664. 28346267 PMC5466356

[R173] Leslie Eva, Sugiyama Takemi, Ierodiaconou Daniel, Kremer Peter, 2010. Perceived and objectively measured greenness of neighbourhoods: are they measuring the same thing? Landsc. Urban Plann 95 (1), 28–33. 10.1016/j.landurbplan.2009.11.002.

[R174] Li Hansen, Browning Matthew H.E. M., Rigolon Alessandro, , 2023. Beyond “Bluespace” and “Greenspace”: a narrative review of possible health benefits from exposure to other natural landscapes. Sci. Total Environ 856 (January), 159292. 10.1016/j.scitotenv.2022.159292. 36208731

[R175] Lim Chris C., Hayes Richard B., Ahn Jiyoung, , 2019. Mediterranean diet and the association between air pollution and cardiovascular disease mortality risk. Circulation 139 (15), 1766–1775. 10.1161/CIRCULATIONAHA.118.035742. 30700142 PMC6453737

[R176] Lindsay Tim, Westgate Kate, Wijndaele Katrien, , 2019. Descriptive epidemiology of physical activity energy expenditure in UK adults (the fenland study). Int. J. Behav. Nutr. Phys. Activ 16 (1), 126. 10.1186/s12966-019-0882-6. PMC6902569 31818302

[R177] Liu Yang, Kwan Mei-Po, Wong Man Sing, Yu Changda, 2023. Current methods for evaluating people’s exposure to green space: a scoping review. Soc. Sci. Med 338 (December), 116303. 10.1016/j.socscimed.2023.116303. 37866172

[R178] Lloyd Simon J., Quijal-Zamorano Marcos, Achebak Hicham, , 2023. The direct and indirect influences of interrelated regional-level sociodemographic factors on heat-attributable mortality in Europe: insights for adaptation strategies. Environ. Health Perspect 131 (8), 087013. 10.1289/EHP11766. 37606292 PMC10443201

[R179] Locher Barbara, Piquerez André, Habermacher Manuel, , 2018. Differences between outdoor and indoor sound levels for open, tilted, and closed windows. Int. J. Environ. Res. Publ. Health 15 (1), 149. 10.3390/ijerph15010149. PMC5800248 29346318

[R180] Loomis Dana, Dzhambov Angel M., Momen Natalie C., , 2022. The effect of occupational exposure to welding fumes on trachea, bronchus and lung cancer: a systematic review and meta-analysis from the WHO/ILO joint estimates of the work-related burden of disease and injury. Environ. Int 170 (December), 107565. 10.1016/j.envint.2022.107565. 36402034

[R181] López-Bueno JA, Navas-Martín MA, Díaz J, , 2022. Analysis of vulnerability to heat in rural and urban areas in Spain: what factors explain heat’s geographic behavior? Environ. Res 207 (May), 112213. 10.1016/j.envres.2021.112213. 34666017

[R182] Lowe Melanie, Adlakha Deepti, Sallis James F., , 2022. City planning policies to support health and sustainability: an international comparison of policy indicators for 25 cities. Lancet Global Health 10 (6), 6. 10.1016/S2214-109X(22)00069-9. PMC9906636 35561723

[R183] Lygum Victoria Linn, Dupret Katia, Bentsen Peter, , 2023. Greenspace as workplace: benefits, challenges and essentialities in the physical environment. Int. J. Environ. Res. Publ. Health 20 (17), 6689. 10.3390/ijerph20176689. PMC10488277 37681829

[R184] Maitre Léa, Guimbaud Jean-Baptiste, Warembourg Charline, , 2022. State-of-the-Art methods for exposure-health studies: results from the exposome data challenge event. Environ. Int 168 (October), 107422. 10.1016/j.envint.2022.107422. 36058017

[R185] Malekzadeh Arianne, Michels Kathleen, Wolfman Celia, Anand Nalini, Sturke Rachel, 2020. Strengthening research capacity in LMICs to address the global NCD burden. Glob. Health Action 13 (1), 1846904. 10.1080/16549716.2020.1846904. 33373280 PMC7782223

[R186] Malmqvist E, Oudin A, Pascal M, Medina S, 2018. Choices behind numbers: a review of the major air pollution health impact assessments in Europe. Curr. Environ. Health Rep 5 (1), 1. 10.1007/s40572-018-0175-2. 29404862 PMC5876343

[R187] Manoli Gabriele, Fatichi Simone, Schläpfer Markus, , 2019. Magnitude of urban heat islands largely explained by climate and population. Nature 573 (7772), 55–60. 10.1038/s41586-019-1512-9. 31485056

[R188] Markevych Iana, Schoierer Julia, Hartig Terry, , 2017. Exploring pathways linking greenspace to health: theoretical and methodological guidance. Environ. Res 158 (October), 301–317. 10.1016/j.envres.2017.06.028. 28672128

[R189] Marselle Melissa R., Hartig Terry, Cox Daniel T.C., , 2021. Pathways linking biodiversity to human health: a conceptual framework. Environ. Int 150 (May), 106420. 10.1016/j.envint.2021.106420. 33556912

[R190] Martinez-Gomez David, Cabanas-Sanchez Veronica, Yu Tsung, , 2022. Long-term leisure-time physical activity and risk of all-cause and cardiovascular mortality: dose-response associations in a prospective cohort study of 210 327 Taiwanese adults. Br. J. Sports Med 56 (16), 919–926. 10.1136/bjsports-2021-104961. 35387777

[R191] Martinez-Gomez David, Ekelund Ulf, Saint-Maurice Pedro F., Cabanas-Sánchez Verónica, 2025. Anticipating key innovations in physical activity epidemiology for the coming decades. Innovation 0 (0). 10.1016/j.xinn.2025.101045. PMC12925899 41737313

[R192] Masselot Pierre, Mistry Malcolm, Vanoli Jacopo, , 2023. Excess mortality attributed to heat and cold: a health impact assessment study in 854 cities in Europe. Lancet Planet. Health 7 (4), e271–e281. 10.1016/S2542-5196(23)00023-2. 36934727

[R193] Masselot Pierre, Kan Haidong, Kharol Shailesh K., , 2024. Air pollution mixture complexity and its effect on PM2.5-Related mortality: a multicountry time-series study in 264 cities. Environmental Epidemiology 8 (6), e342. 10.1097/EE9.0000000000000342. 39483640 PMC11527422

[R194] Masselot Pierre, Mistry Malcolm N., Rao Shilpa, , 2025. Estimating future heat-related and cold-related mortality under climate change, demographic and adaptation scenarios in 854 European cities. Nat. Med 31 (4), 1294–1302. 10.1038/s41591-024-03452-2. 39870815 PMC12003192

[R195] McDuffie Erin E., Smith Steven J., Rourke Patrick O., , 2020. A global anthropogenic emission inventory of atmospheric pollutants from Sector- and fuel-specific sources (1970–2017): an application of the community emissions data system (CEDS). Earth Syst. Sci. Data 12 (4), 3413–3442. 10.5194/essd-12-3413-2020.

[R196] McDuffie Erin E., Martin Randall V., Spadaro Joseph V., , 2021. Source sector and fuel contributions to ambient PM2.5 and attributable mortality across multiple spatial scales. Nat. Commun 12 (1), 3594. 10.1038/s41467-021-23853-y. 34127654 PMC8203641

[R197] Menon JML, Struijs F, Whaley P, 2022. The methodological rigour of systematic reviews in environmental health. Crit. Rev. Toxicol 52 (3), 3. 10.1080/10408444.2022.2082917. 35793403

[R198] Mitchell Richard, Astell-Burt Thomas, Richardson Elizabeth A., 2011. A comparison of green space indicators for epidemiological research. Research Report. J Epidemiol Community Health 65 (10), 853–858. 10.1136/jech.2010.119172. 21296907

[R199] Mitsakou Christina, Dimitroulopoulou Sani, Heaviside Clare, , 2019. Environmental public health risks in European metropolitan areas within the EURO-HEALTHY project. Sci. Total Environ 658 (March), 1630–1639. 10.1016/j.scitotenv.2018.12.130. 30678019

[R200] Moser Susanne C., Hart Juliette A. Finzi, 2015. The long arm of climate change: societal teleconnections and the future of climate change impacts studies. Clim. Change 129 (1), 13–26. 10.1007/s10584-015-1328-z. 32214560 PMC7088147

[R201] Mueller Natalie, Rojas-Rueda David, Basagaña Xavier, , 2017. Urban and transport planning related exposures and mortality: a health impact assessment for cities. Environ. Health Perspect 125 (1), 89–96. 10.1289/EHP220. 27346385 PMC5226698

[R202] Mueller Natalie, Rojas-Rueda David, Salmon Maëlle, , 2018. Health impact assessment of cycling network expansions in European cities. Prev. Med 109 (April), 62–70. 10.1016/j.ypmed.2017.12.011. 29330030

[R203] Mueller Natalie, Anderle Rodrigo, Brachowicz Nicolai, , 2023. Model choice for quantitative health impact assessment and modelling: an expert consultation and narrative literature review. Int. J. Health Pol. Manag 12 (March), 7103. 10.34172/ijhpm.2023.7103. PMC10461835 37579425

[R204] Murray Christopher J.L., 2022. The global burden of disease study at 30 years. Nat. Med 28 (10), 2019–2026. 10.1038/s41591-022-01990-1. 36216939

[R205] Nachman Keeve E., 2011. Leveraging epidemiology to improve risk assessment. Open Epidemiol. J 4 (1), 3–29. 10.2174/1874297101104010003. 31341519 PMC6655421

[R206] Nazarian Negin, Lee Jason KW., 2021. Personal assessment of urban heat exposure: a systematic review. Environ. Res. Lett 16 (3), 033005. 10.1088/1748-9326/abd350.

[R207] Ndahimana Didace, Kim Eun-Kyung, 2017. Measurement methods for physical activity and energy expenditure: a review. Clinical Nutrition Research 6 (2), 68–80. 10.7762/cnr.2017.6.2.68. 28503503 PMC5426207

[R208] Nethery Rachel C., Dominici Francesca, 2019. Estimating pollution-attributable mortality at the regional and global scales: challenges in uncertainty estimation and causal inference. Eur. Heart J 40 (20), 1597–1599. 10.1093/eurheartj/ehz200. 31004133 PMC6528153

[R209] Nguyen Phi-Yen, Astell-Burt Thomas, Rahimi-Ardabili Hania, Feng Xiaoqi, 2021. Green space quality and health: a systematic review. Int. J. Environ. Res. Publ. Health 18 (21), 11028. 10.3390/ijerph182111028. PMC8582763 34769549

[R210] Nieuwenhuijsen Mark J., 2020. Urban and transport planning pathways to carbon neutral, liveable and healthy cities; A review of the current evidence. Environ. Int 140 (July), 105661. 10.1016/j.envint.2020.105661. 32307209

[R211] Nieuwenhuijsen Mark J., 2021. New urban models for more sustainable, liveable and healthier cities post Covid19; reducing air pollution, noise and heat island effects and increasing green space and physical activity. Environ. Int 157 (December), 106850. 10.1016/j.envint.2021.106850. 34531034 PMC8457623

[R212] Nieuwenhuijsen Mark J., Khreis Haneen, Verlinghieri Ersilia, Mueller Natalie, Rojas-Rueda David, 2017. Participatory quantitative health impact assessment of urban and transport planning in cities: a review and research needs. Environ. Int 103 (June), 61–72. 10.1016/j.envint.2017.03.022. 28389127

[R213] Nieuwenhuijsen Mark J., Gascon Mireia, Martinez David, , 2018. Air pollution, noise, blue space, and green space and premature mortality in Barcelona: a mega cohort. Int. J. Environ. Res. Publ. Health 15 (11), 2405. 10.3390/ijerph15112405. PMC6265844 30380717

[R214] Nieuwenhuijsen Mark, Khreis Haneen, Verlinghieri Ersilia, Mueller Natalie, Rojas-Rueda David, 2019. The role of health impact assessment for shaping policies and making cities healthier. In: Nieuwenhuijsen Mark, Khreis Haneen (Eds.), Integrating Human Health into Urban and Transport Planning: a Framework. Springer International Publishing. 10.1007/978-3-319-74983-9_29.

[R215] Nieuwenhuijsen Mark, De Nazelle Audrey, Garcia-Aymerich Judith, Khreis Haneen, Hoffmann Barbara, 2024. Shaping urban environments to improve respiratory health: recommendations for research, planning, and policy. Lancet Respir. Med 12 (3), 247–254. 10.1016/S2213-2600(23)00329-6. 37866374

[R216] Nobile Federica, Dimakopoulou Konstantina, Åström Christofer, , 2024. External exposome and all-cause mortality in European cohorts: the EXPANSE project. Frontiers in Epidemiology 4 (May), 1327218. 10.3389/fepid.2024.1327218. 38863881 PMC11165119

[R217] Noor Jolly, Bezgrebelna Mariya, Kerman Nick, , 2025. Heat-related health risks for people experiencing homelessness: a rapid review. J. Urban Health 102 (2), 305–331. 10.1007/s11524-025-00968-x. 40106210 PMC12031682

[R218] Ntarladima Anna-Maria, Karssenberg Derek, Vaartjes Ilonca, , 2021. A comparison of associations with childhood lung function between air pollution exposure assessment methods with and without accounting for time-activity patterns. Environ. Res 202 (November), 111710. 10.1016/j.envres.2021.111710. 34280420

[R219] Odebeatu Chinonso Christian, Darsy Darssan, Roscoe Charlotte, Ahmed Muktar, Simon Reid, Osborne Nicholas J., 2024. Greenspace and risk of obesity-related cancer in the UK biobank cohort: an analysis of private residential gardens and other greenspace types. Sci. Total Environ 943 (September), 173833. 10.1016/j.scitotenv.2024.173833. 38866159

[R220] OECD, 2019. The Economic Cost of Air Pollution: Evidence from Europe’. OECD. December 12. https://www.oecd.org/en/publications/the-economic-cost-of-air-pollution-evidence-from-europe_56119490-en.html.

[R221] OHAT, 2019. Handbook for Conducting a Literature-based Health Assessment Using OHAT Approach for Systematic Review and Evidence Integration.

[R222] Oreggioni GD, Mahiques O, Monforti-Ferrario F, , 2022. The impacts of technological changes and regulatory frameworks on global air pollutant emissions from the energy industry and road transport. Energy Policy 168 (September), 113021. 10.1016/j.enpol.2022.113021.

[R223] Orellano Pablo, Kasdagli Maria-Iosifina, Velasco Román Pérez, Samoli Evangelia, 2024. Long-term exposure to particulate matter and mortality: an update of the WHO global air quality guidelines systematic review and meta-analysis. Int. J. Publ. Health 69 (September), 1607683. 10.3389/ijph.2024.1607683. PMC11466858 39399882

[R224] Orru Hans, Raza Wasif, Forastiere Francesco, , 2025. A Review of the Evidence of the Toxicity of Chemical Substances Included in the European Union Ambient Air Quality and Drinking Water Directives: Perspectives for Health Impact Assessments. Environment & Health. 10.1021/envhealth.4c00277 ahead of print, July 1. PMC12455347 40995490

[R225] Osei Francis, Effah Esther, 2022. Health effects caused by noise - the case of Africa: evidence in literature from the past 25 years. Asian Journal of Advanced Research and Reports, March 19–27. 10.9734/AJARR/2022/v16i230452.

[R226] PAGAC, 2018. 2018 physical activity guidelines advisory committee scientific report. https://odphp.health.gov/our-work/nutrition-physical-activity/physical-activity-guidelines/current-guidelines/scientific-report.

[R227] Pascal Mathilde, Chanel Perrine de Crouy, Wagner Verene, , 2016. The mortality impacts of fine particles in France. Sci. Total Environ 571, 416–425. 10.1016/j.scitotenv.2016.06.213. 27453142

[R228] Pearce Matthew, Bishop Tom R.P., Sharp Stephen, , 2020. Network harmonization of physical activity variables through indirect validation. Journal for the Measurement of Physical Behaviour. March 1. 10.1123/jmpb.2019-0001.

[R229] Pearce Matthew, Garcia Leandro, Ali Abbas, , 2022. Association between physical activity and risk of depression: a systematic review and meta-analysis. JAMA Psychiatry 79 (6), 550–559. 10.1001/jamapsychiatry.2022.0609. 35416941 PMC9008579

[R230] Périard Julien D., Eijsvogels Thijs M.H., Daanen Hein A.M., 2021. Exercise under heat stress: thermoregulation, hydration, performance implications, and mitigation strategies. Physiol. Rev 101 (4), 1873–1979. 10.1152/physrev.00038.2020. 33829868

[R231] Pershagen Göran, Pyko Andrei, Marit Aasvang Gunn, , 2025. Road traffic noise and incident ischemic heart disease, myocardial infarction, and stroke: a systematic review and meta-analysis. Environmental Epidemiology 9 (3), e400. 10.1097/EE9.0000000000000400. 40444274 PMC12122180

[R232] Piggin Joe, 2020. What is physical activity? A holistic definition for teachers, researchers and policy makers. Frontiers in Sports and Active Living 2 (June). 10.3389/fspor.2020.00072. PMC7739796 33345063

[R233] Pozzer A, Anenberg SC, Dey S, Haines A, Lelieveld J, Chowdhury S, 2023. Mortality attributable to ambient air pollution: a review of global estimates. GeoHealth 7 (1), 1. 10.1029/2022GH000711. PMC9828848 36636746

[R234] Prince Stephanie A., Lund Rasmussen Charlotte, Biswas Aviroop, , 2021. The effect of leisure time physical activity and sedentary behaviour on the health of workers with different occupational physical activity demands: a systematic review. Int. J. Behav. Nutr. Phys. Activ 18 (1), 100. 10.1186/s12966-021-01166-z. PMC8290554 34284795

[R235] Quinn Tyler D., Gibbs Bethany Barone, 2023. Context matters: the importance of physical activity domains for public health. Journal for the Measurement of Physical Behaviour. October 4. 10.1123/jmpb.2023-0030.

[R236] Ragavan Maya I., Marcil Lucy E., Garg Arvin, 2020. Climate change as a social determinant of health. Pediatrics 145 (5), e20193169. 10.1542/peds.2019-3169. 32265296 PMC7193972

[R237] Rai Masna, Breitner Susanne, Zhang Siqi, Rappold Ana G., Schneider Alexandra, 2022. Achievements and gaps in projection studies on the temperature-attributable health burden: where should we be headed? Frontiers in Epidemiology 2 (December). 10.3389/fepid.2022.1063871. PMC10631562 37942471

[R238] Neimann Rasmussen Lauge, Montgomery Paul, 2018. The prevalence of and factors associated with inclusion of non-english language studies in campbell systematic reviews: a survey and meta-epidemiological study. Syst. Rev 7 (1), 129. 10.1186/s13643-018-0786-6. 30139391 PMC6107944

[R239] Rigaud Maxime, Buekers Jurgen, Bessems Jos, , 2024. The methodology of quantitative risk assessment studies. Environmental Health 23 (1), 13. 10.1186/s12940-023-01039-x. 38281011 PMC10821313

[R240] Rogowski Clare B. Best, Bredell Christiaan, Shi Yan, , 2025. Long-term air pollution exposure and incident dementia: a systematic review and meta-analysis. Lancet Planet. Health 9 (7). 10.1016/S2542-5196(25)00118-4. 40716448

[R241] Rojas-Rueda David, Nieuwenhuijsen Mark J., Gascon Mireia, Perez-Leon Daniela, Mudu Pierpaolo, 2019. Green spaces and mortality: a systematic review and meta-analysis of cohort studies. Lancet Planet. Health 3 (11), 11. 10.1016/S2542-5196(19)30215-3. PMC6873641 31777338

[R242] Rojas-Rueda David, Vaught Elida, Buss Daniel, 2021. Why a new research agenda on green spaces and health is needed in Latin America: results of a systematic review. Int. J. Environ. Res. Publ. Health 18 (11), 11. 10.3390/ijerph18115839 rayyan-714274959. PMC8198896 34072319

[R243] Ronaldson Amy, Arias de la Torre Jorge, Ashworth Mark, , 2022. Associations between air pollution and multimorbidity in the UK biobank: a cross-sectional study. Front. Public Health 10, 1035415. 10.3389/fpubh.2022.1035415. 36530697 PMC9755180

[R244] Roscoe Charlotte, Grady Stephanie T., Hart Jaime E., , 2023. Association between noise and cardiovascular disease in a nationwide U.S. prospective cohort study of women followed from 1988 to 2018. Environ. Health Perspect 131 (12), 127005. 10.1289/EHP12906. 38048103 PMC10695265

[R245] Ross Robert, Janssen Ian, Tremblay Mark S., 2024. Public health importance of light intensity physical activity. Journal of Sport and Health Science 13 (5), 674–675. 10.1016/j.jshs.2024.01.010. 38307207 PMC11282331

[R246] Ru Muye, Shindell Drew, Spadaro Joseph V., , 2023. New concentration-response functions for seven morbidity endpoints associated with short-term PM2.5 exposure and their implications for health impact assessment. Environ. Int 179 (September), 108122. 10.1016/j.envint.2023.108122. 37659174

[R247] Rugel Emily Jessica, Brauer Michael, 2020. Quiet, clean, green, and active: a navigation guide systematic review of the impacts of spatially correlated urban exposures on a range of physical health outcomes. Environ. Res 185, 109388. 10.1016/j.envres.2020.109388. 32244108

[R248] Sakhvidi Zare, Javad Mohammad, Mehrparvar Amir Houshang, Sakhvidi Fariba Zare, Dadvand Payam, 2023. Greenspace and health, wellbeing, physical activity, and development in children and adolescents: an overview of the systematic reviews. Current Opinion in Environmental Science & Health 32 (April), 100445. 10.1016/j.coesh.2023.100445.

[R249] Samoli Evangelia, Butland Barbara K., 2017. Incorporating measurement error from modeled air pollution exposures into epidemiological analyses. Curr. Environ. Health Rep 4 (4), 472–480. 10.1007/s40572-017-0160-1. 28983855

[R250] Savitz David A., Hattersley Anne M., 2023. Evaluating chemical mixtures in epidemiological studies to inform regulatory decisions. Environ. Health Perspect 131 (4), 045001. 10.1289/EHP11899. 37022726 PMC10078806

[R251] Schroeder Anna, Tatah Lambed, Ali Abbas, , 2025. Uncertainty and value of information analysis in the integrated transport and health impact modelling tool for global cities (ITHIM-Global). Preprint, medRxiv. 10.1101/2025.08.15.25333754. August 17.

[R252] Scovronick Noah, Sera Francesco, Vu Bryan, , 2024. Temperature-mortality associations by age and cause: a multi-country multi-city study. Environmental Epidemiology 8 (5), 5. 10.1097/EE9.0000000000000336. PMC11424137 39323989

[R253] Seidler Andreas, Hegewald Janice, Seidler Anna Lene, Schubert Melanie, Zeeb Hajo, 2019. Is the whole more than the sum of its parts? Health effects of different types of traffic noise combined. Int. J. Environ. Res. Publ. Health 16 (9), 1665. 10.3390/ijerph16091665. PMC6539743 31086115

[R254] Senerth Emily, Tangri Neha, Krammer Lori, , 2024. Systematic review methods in environmental health: a critical interpretive synthesis to inform the evolution of systematic review guidance. Evidence-Based Toxicology 2 (1), 2443410. 10.1080/2833373X.2024.2443410.

[R255] Sera Francesco, Armstrong Ben, Tobias Aurelio, , 2019. How urban characteristics affect vulnerability to heat and cold: a multi-country analysis. Int. J. Epidemiol 48 (4), 1101–1112. 10.1093/ije/dyz008. 30815699

[R256] Shaffer Rachel M., Sellers Samuel P., Baker Marissa G., , 2019. Improving and expanding estimates of the global burden of disease due to environmental health risk factors. Environ. Health Perspect 127 (10), 105001. 10.1289/EHP5496. 31626566 PMC6867191

[R257] Shaffer Rachel M., Lee Alexandra L., Nachman Rebecca, Christensen Krista, Bateson Thomas F., 2025. A perspective from US environmental protection agency (EPA) scientists: how your epidemiologic analyses can inform the human health risk assessment process. Environ. Health Perspect 133 (3–4), 045001. 10.1289/EHP15203. 40048177 PMC12010935

[R258] Shan Xiaorong, Casey Joan A., Shearston Jenni A., Henneman Lucas R.F., 2024. Methods for quantifying source-specific air pollution exposure to serve epidemiology, risk assessment, and environmental justice. GeoHealth 8 (11), e2024GH001188. 10.1029/2024GH001188. PMC11536408 39502358

[R259] SheikhMozafari Mohammad Javad, Shekaftik Soqrat Omari, Fasih Ramandi Fatameh, Esmaeelpour Mohammad Reza Monazzam, Biganeh Jamal, 2025. A review of the studies investigating the effects of noise exposure on humans from 2017 to 2022: trends and knowledge gaps. Noise Mapp. 12 (1). 10.1515/noise-2025-0015.

[R260] Sheppard Lianne, Burnett Richard T., Szpiro Adam A., , 2012. Confounding and exposure measurement error in air pollution epidemiology. Air Qual. Atmos. Health 5 (2), 203–216. 10.1007/s11869-011-0140-9. 22662023 PMC3353104

[R261] Shi Xiaoting, Wallach Joshua D., 2022. Umbrella reviews: a useful study design in need of standardisation. BMJ 5, o1740. 10.1136/bmj.o1740.

[R262] Shojania Kaveh G., Sampson Margaret, Ansari Mohammed T., Ji Jun, Doucette Steve, Moher David, 2007. How quickly do systematic reviews Go out of date? A survival analysis. Ann. Intern. Med 147 (4), 224–233. 10.7326/0003-4819-147-4-200708210-00179. 17638714

[R263] Sillman Delaney, Rigolon Alessandro, Browning Matthew H.E. M., Yoon Hyunseo Violet, McAnirlin Olivia, 2022. Do sex and gender modify the association between green space and physical health? A systematic review. Environ. Res 209, 112869. 10.1016/j.envres.2022.112869. 35123971

[R264] Singh Nidhi, Areal Ashtyn Tracy, Breitner Susanne, , 2024. Heat and cardiovascular mortality: an epidemiological perspective. Circ. Res 134 (9), 1098–1112. 10.1161/CIRCRESAHA.123.323615. 38662866 PMC11042530

[R265] Smith James David, Mitsakou Christina, Kitwiroon Nutthida, , 2016. London hybrid exposure model: improving human exposure estimates to NO _2_ and PM _2.5_ in an urban setting. Environmental Science & Technology 50 (21), 21. 10.1021/acs.est.6b01817. 27706935

[R266] Sohrabi Soheil, Khreis Haneen, 2020. Burden of disease from transportation noise and motor vehicle crashes: analysis of data from Houston, Texas. Environ. Int 136 (March), 105520. 10.1016/j.envint.2020.105520. 32044176

[R267] Son JY, Liu JC, Bell ML, 2019. Temperature-related mortality: a systematic review and investigation of effect modifiers. Environ. Res. Lett 14 (7), 7. 10.1088/1748-9326/ab1cdb. PMC12362558 40837670

[R268] Song Jinglu, Gasparrini Antonio, Wei Di, , 2024. Do greenspaces really reduce heat health impacts? Evidence for different vegetation types and distance-based greenspace exposure. Environ. Int 191 (September), 108950. 10.1016/j.envint.2024.108950. 39190977

[R269] Sørensen Mette, Pershagen Göran, Thacher Jesse Daniel, , 2023. Health position paper and redox perspectives - disease burden by transportation noise. Redox Biol. 69 (December), 102995. 10.1016/j.redox.2023.102995. 38142584 PMC10788624

[R270] Stafoggia Massimo, Breitner Susanne, Hampel Regina, Basagaña Xavier, 2017. Statistical approaches to address multi-pollutant mixtures and multiple exposures: the state of the science. Curr. Environ. Health Rep 4 (4), 481–490. 10.1007/s40572-017-0162-z. 28988291

[R271] Stafoggia Massimo, Oftedal Bente, Chen Jie, , 2022. Long-term exposure to low ambient air pollution concentrations and mortality among 28 million people: results from seven large European cohorts within the ELAPSE project. The Lancct. Planetary Health 6 (1), e9–e18. 10.1016/S2542-5196(21)00277-1. 34998464

[R272] Stafoggia Massimo, Michelozzi Paola, Schneider Alexandra, , 2023. Joint effect of heat and air pollution on mortality in 620 cities of 36 countries. Environ. Int 181 (November), 108258. 10.1016/j.envint.2023.108258. 37837748 PMC10702017

[R273] Staves Corin, Zhang Qin, Moeckel Rolf, Woodcock James, 2023. Integrating health effects within an agent-based land use and transport model. J. Transport Health 33 (November), 101707. 10.1016/j.jth.2023.101707.

[R274] Steenland K, Straif K, Schubauer-Berigan MK, , 2025. Letter: robins-E risk of bias tool. Environ. Int 199 (May), 109463. 10.1016/j.envint.2025.109463. 40307162

[R275] Strain Tessa, Wijndaele Katrien, Dempsey Paddy C., , 2020. Wearable-device-measured physical activity and future health risk. Nat. Med 26 (9), 1385–1391. 10.1038/s41591-020-1012-3. 32807930 PMC7116559

[R276] Strain Tessa, Wijndaele Katrien, Pearce Matthew, Brage Søren, 2022. Considerations for the use of consumer-grade wearables and smartphones in population surveillance of physical activity. Journal for the Measurement of Physical Behaviour. February 2. 10.1123/jmpb.2021-0046.

[R277] Strak M, Weinmayr G, Rodopoulou S, Chen J, De Hoogh K, Andersen ZJ, Atkinson R, Bauwelinck M, Bekkevold T, Bellander T, Boutron-Ruault MC, 2021. Long term exposure to low level air pollution and mortality in eight European cohorts within the ELAPSE project: pooled analysis. bmj 374. 10.1136/bmj.n1904 PMC8409282 34470785

[R278] Sutton Patrice, Chartres Nicholas, Rayasam Swati D.G., , 2021. Reviews in environmental health: how systematic are they? Environ. Int 152 (July), 106473. 10.1016/j.envint.2021.106473. 33798823 PMC8118386

[R279] Tainio Marko, de Nazelle Audrey J., Götschi Thomas, , 2016. Can air pollution negate the health benefits of cycling and walking? Prev. Med 87 (June), 233–236. 10.1016/j.ypmed.2016.02.002. 27156248 PMC4893018

[R280] Tainio Marko, Andersen Zorana Jovanovic, Nieuwenhuijsen Mark J., , 2021. Air pollution, physical activity and health: a mapping review of the evidence. Environ. Int 147 (February), 105954. 10.1016/j.envint.2020.105954. 33352412 PMC7816214

[R281] Taylor Lucy, Hochuli Dieter F., 2017. Defining greenspace: multiple uses across multiple disciplines. Landsc. Urban Plann 158 (February), 25–38. 10.1016/j.landurbplan.2016.09.024.

[R282] Teixeira Liliane R., Frank Pega, Dzhambov Angel M., , 2021. The effect of occupational exposure to noise on ischaemic heart disease, stroke and hypertension: a systematic review and meta-analysis from the WHO/ILO joint estimates of the work-related burden of disease and injury. Environ. Int 154 (September), 106387. 10.1016/j.envint.2021.106387. 33612311 PMC8204276

[R283] The Academy of Medical Sciences, 2018. ‘Multiple long-term conditions (Multimorbidity): a priority for global health research’. April. https://acmedsci.ac.uk/policy/policy-projects/multimorbidity.

[R284] Thondoo M, Goel R, Tatah L, Naraynen N, Woodcock J, Nieuwenhuijsen Mark, 2022. The built environment and health in Low- and middle-income countries: a review on quantitative health impact assessments. Curr. Environ. Health Rep 9 (1), 90–103. 10.1007/s40572-021-00324-6. 34514535

[R285] Tobías Aurelio, Hashizume Masahiro, Honda Yasushi, , 2021. Geographical variations of the minimum mortality temperature at a global scale: a multicountry study. Environmental Epidemiology (Philadelphia, Pa.) 5 (5), e169. 10.1097/EE9.0000000000000169. 34934890 PMC8683148

[R286] Tonne Cathryn, Basagaña Xavier, Chaix Basile, , 2017. New frontiers for environmental epidemiology in a changing world. Environ. Int 104 (July), 155–162. 10.1016/j.envint.2017.04.003. 28454882

[R287] Tonne Cathryn, Adair Linda, Adlakha Deepti, , 2021. Defining pathways to healthy sustainable urban development. Environ. Int 146 (January), 106236. 10.1016/j.envint.2020.106236. 33161201

[R288] Tran Phuong Bich, Kazibwe Joseph, Nikolaidis Georgios F., Linnosmaa Ismo, Rijken Mieke, van Olmen Josefien, 2022. Costs of multimorbidity: a systematic review and meta-analyses. BMC Med. 20 (1), 234. 10.1186/s12916-022-02427-9. 35850686 PMC9295506

[R289] Troiano Richard P., McClain James J., Brychta Robert J., Chen Kong Y., 2014. ‘Evolution of accelerometer methods for physical activity research’. Review. Br. J. Sports Med 48 (13), 1019–1023. 10.1136/bjsports-2014-093546. 24782483 PMC4141534

[R290] Ueberham Maximilian, Schlink Uwe, 2018. Wearable sensors for multifactorial personal exposure measurements – a ranking study. Environ. Int 121 (December), 130–138. 10.1016/j.envint.2018.08.057. 30199668

[R291] UK Health Security Agency, 2024. Chapter 2. Temperature Effects on Mortality in a Changing Climate UK Health Security Agency. January 15. https://www.gov.uk/government/publications/climate-change-health-effects-in-the-uk.

[R292] US EPA, 2017. The health impact assessment (HIA) resource and tool compilation. Overviews and Factsheets. July 19. https://www.epa.gov/healthresearch/health-impact-assessment-hia-resource-and-tool-compilation.

[R293] US EPA, 2019. Integrated science assessment (ISA) for particulate matter (final report, Dec 2019). Reports & Assessments. https://cfpub.epa.gov/ncea/isa/recordisplay.cfm?deid=347534.

[R294] Vanos Jennifer K., Baldwin Jane W., Jay Ollie, Ebi Kristie L., 2020. Simplicity lacks robustness when projecting heat-health outcomes in a changing climate. Nat. Commun 11 (1), 6079. 10.1038/s41467-020-19994-1. 33247118 PMC7695704

[R295] Vicedo-Cabrera AM, Scovronick N, Sera F, , 2021. The burden of heat-related mortality attributable to recent human-induced climate change. Nat. Clim. Change 11 (6), 492–500. 10.1038/s41558-021-01058-x. PMC7611104 34221128

[R296] Vienneau Danielle, Wunderli Jean Marc, 2023. Invited perspective: cutting through the Noise—The national park service anthropogenic noise model for exposure assessment. Environ. Health Perspect 131 (12), 121304. 10.1289/EHP14056. 38048102 PMC10695264

[R297] Vienneau Danielle, Héritier Harris, Foraster Maria, , 2019. Façades, floors and maps – influence of exposure measurement error on the association between transportation noise and myocardial infarction. Environ. Int 123 (February), 399–406. 10.1016/j.envint.2018.12.015. 30622064

[R298] Vilcins Dwan, Christofferson Rebecca C., Yoon Jin-Ho, , 2024a. Updates in air pollution: current research and future challenges. Ann. Glob. Health 90 (1), 1. 10.5334/aogh.4363. 38312715 PMC10836163

[R299] Vilcins Dwan, Sly Peter D., Scarth Peter, Mavoa Suzanne, 2024b. Green space in health research: an overview of common indicators of greenness. Rev. Environ. Health 39 (2), 221–231. 10.1515/reveh-2022-0083. 36372560

[R300] Vodonos Alina, Abu Awad Yara, Schwartz Joel, 2018. The concentration-response between long-term PM2.5 exposure and mortality; A meta-regression approach. Environ. Res 166, 677–689. 10.1016/j.envres.2018.06.021. 30077140

[R301] Wang Huaqing, Gholami Simin, Xu Wenyan, Samavatekbatan Amirhossein, Sleipness Ole, Tassinary Louis G., 2024. Where and how to invest in greenspace for optimal health benefits: a systematic review of greenspace morphology and human health relationships. Lancet Planet. Health 8 (8), e574–e587. 10.1016/S2542-5196(24)00140-2. 39122326

[R302] Warren Janet M., Ekelund Ulf, Besson Herve, , 2010. Assessment of physical activity - a review of methodologies with reference to epidemiological research: a report of the exercise physiology section of the European association of cardiovascular prevention and rehabilitation. Eur. J. Cardiovasc. Prev. Rehabil.: Official Journal of the European Society of Cardiology, Working Groups on Epidemiology & Prevention and Cardiac Rehabilitation and Exercise Physiology 17 (2), 127–139. 10.1097/HJR.0b013e32832ed875. 20215971

[R303] Wei Yaguang, Qiu Xinye, Yazdi Mahdieh Danesh, , 2022. The impact of exposure measurement error on the estimated concentration–response relationship between long-term exposure to PM2.5 and mortality. Environ. Health Perspect 130 (7), 077006. 10.1289/EHP10389. 35904519 PMC9337229

[R304] Weichenthal Scott, Pinault Lauren L., Burnett Richard T., 2017. Impact of oxidant gases on the relationship between outdoor fine particulate air pollution and nonaccidental, cardiovascular, and respiratory mortality. Sci. Rep 7 (November), 16401. 10.1038/s41598-017-16770-y. 29180643 PMC5703979

[R305] Weichenthal Scott, Pinault Lauren, Christidis Tanya, , 2022. How low can you go? Air pollution affects mortality at very low levels. Sci. Adv 8 (39), eabo3381. 10.1126/sciadv.abo3381. PMC9519036 36170354

[R306] Welk Gregory J., Yang Bai, Lee Jung-Min, Godino Job, Saint-Maurice Pedro F., Carr Lucas, 2019. Standardizing analytic methods and reporting in activity monitor validation studies. Med. Sci. Sports Exerc 51 (8), 1767–1780. 10.1249/MSS.0000000000001966. 30913159 PMC6693923

[R307] Whaley Paul, Halsall Crispin, Ågerstrand Marlene, , 2016. Implementing systematic review techniques in chemical risk assessment: challenges, opportunities and recommendations. Environ. Int 92 (93), 556–564. 10.1016/j.envint.2015.11.002. 26687863 PMC4881816

[R308] White Mathew P., Alcock Ian, Grellier James, , 2019. Spending at least 120 minutes a week in nature is associated with good health and wellbeing. Sci. Rep 9 (June), 7730. 10.1038/s41598-019-44097-3. 31197192 PMC6565732

[R309] White Mathew P., Elliott Lewis R., Gascon Mireia, Roberts Bethany, Fleming Lora E., 2020. Blue space, health and well-being: a narrative overview and synthesis of potential benefits. Environ. Res 191 (December), 110169. 10.1016/j.envres.2020.110169. 32971082

[R310] White Mathew P., Elliott Lewis R., Grellier James, , 2021. Associations between green/blue spaces and mental health across 18 countries. Sci. Rep 11 (1), 8903. 10.1038/s41598-021-87675-0. 33903601 PMC8076244

[R311] White Mathew P., Hartig Terry, Martin Leanne, , 2023. Nature-based biopsychosocial resilience: an integrative theoretical framework for research on nature and health. Environ. Int 181 (November), 108234. 10.1016/j.envint.2023.108234. 37832260

[R312] WHO, 2016. Health Risk Assessment of Air Pollution: General Principles. WHO Regional Office for Europe, Copenhagen, 2016. https://www.who.int/publications/i/item/9789289051316.

[R313] WHO, 2018. Environmental Noise Guidelines for the European Region. World Health Organization, Regional Office for Europe. https://www.who.int/europe/publications/i/item/9789289053563.

[R314] WHO, 2020. WHO Guidelines on Physical Activity and Sedentary Behaviour, 2020. World Health Organization, Geneva. https://www.who.int/publications/i/item/9789240015128.

[R315] WHO, 2021. ‘WHO Global Air Quality Guidelines: particulate Matter (PM2.5 and PM10), Ozone, Nitrogen Dioxide, Sulfur Dioxide and Carbon Monoxide’. World Health Organization, Geneva. https://www.who.int/publications/i/item/9789240034228. 34662007

[R316] WHO, 2023. Generating and Working with Evidence for Urban Health: Policy Brief. World Health Organization, Geneva, 2023. Licence: CC BY-NC-SA 3.0 IGO. World Health Organization. https://www.who.int/publications/i/item/9789240084032.

[R317] WHO, 2025. Health Impact Assessments. https://www.who.int/tools/health-impact-assessments.

[R318] Wicki Benedikt, Flückiger Benjamin, Vienneau Danielle, de Hoogh Kees, Röösli Martin, Ragettli Martina S., 2024. Socio-environmental modifiers of heat-related mortality in eight Swiss cities: a case time series analysis. Environ. Res 246 (April), 118116. 10.1016/j.envres.2024.118116. 38184064

[R319] Wild Christopher Paul, 2005. Complementing the genome with an “exposome”: the outstanding challenge of environmental exposure measurement in molecular epidemiology. Cancer Epidemiol. Biomarkers Prev.: A Publication of the American Association for Cancer Research, Cosponsored by the American Society of Preventive Oncology 14 (8), 1847–1850. 10.1158/1055-9965.EPI-05-0456. 16103423

[R320] Willis Mary D., Casey Joan A., Buonocore Jonathan J., 2024. Potential health hazards of cryptocurrency mining: protecting health in a “Digital Oil Boom”. JAMA 332 (16), 1329–1330. 10.1001/jama.2024.15917. 39325478

[R321] Wilt Grete E., Roscoe Charlotte J., Hu Cindy R., , 2023. Minute level smartphone derived exposure to greenness and consumer wearable derived physical activity in a cohort of US women. Environ. Res 237 (November), 116864. 10.1016/j.envres.2023.116864. 37648192 PMC11146007

[R322] Woodcock James, Tatah Lambed, Anciaes Paulo, , 2025. Quantitative health impact assessment of environmental exposures linked to urban transport and land use in Europe: state of practice and research agenda. August 26. 10.17863/CAM.120689. PMC12540632 41118069

[R323] Woodruff Tracey J., Sutton Patrice, 2014. The navigation guide systematic review methodology: a rigorous and transparent method for translating environmental health science into better health outcomes. Environ. Health Perspect 122 (10), 1007–1014. 10.1289/ehp.1307175. 24968373 PMC4181919

[R324] Wunderli Jean Marc, Pieren Reto, Habermacher Manuel, , 2016. Intermittency ratio: a metric reflecting short-term temporal variations of transportation noise exposure. J. Expo. Sci. Environ. Epidemiol 26 (6), 575–585. 10.1038/jes.2015.56. 26350982 PMC5071543

[R325] Xia Xi, Chan Ka Hung, Niu Yue, , 2024. Modelling personal temperature exposure using household and outdoor temperature and questionnaire data: implications for epidemiological studies. Environ. Int 192 (October), 109060. 10.1016/j.envint.2024.109060. 39401479 PMC7616742

[R326] Xiao Christina, Scales James, Chavda Jasmine, , 2024. Children’s health in London and luton (CHILL) cohort: a 12-Month natural experimental study of the effects of the ultra low emission zone on children’s travel to school. Int. J. Behav. Nutr. Phys. Activ 21 (1), 89. 10.1186/s12966-024-01621-7. PMC11375866 39232801

[R327] Xu Rongbin, Ye Tingting, Yue Xu, , 2023. Global population exposure to landscape fire air pollution from 2000 to 2019. Nature 621 (7979), 521–529. 10.1038/s41586-023-06398-6. 37730866 PMC10511322

[R328] Yang Bo-Yi, Zhao Tianyu, Hu Li-Xin, , 2021. Greenspace and human health: an umbrella review. Innovation 2 (4), 4. 10.1016/j.xinn.2021.100164. PMC8479545 34622241

[R329] Yen Hsin Yen, Chiu Huei Ling, Huang Hao Yun, 2021. Green and blue physical activity for quality of life: a systematic review and meta-analysis of randomized control trials. Landsc. Urban Plann 212 (104093). 10.1016/j.landurbplan.2021.104093.

[R330] Yi Li, Hart Jaime E., Roscoe Charlotte, , 2025. Greenspace and depression incidence in the US-Based nationwide nurses’ health study II: a deep learning analysis of street-view imagery. Environ. Int 198 (April), 109429. 10.1016/j.envint.2025.109429. 40209395 PMC12224280

[R331] Yin Jie, Bratman Gregory N., Browning Matthew H.E.M., Spengler John D., Olvera-Alvarez Hector A., 2022. Stress recovery from virtual exposure to a brown (desert) environment versus a green environment. J. Environ. Psychol 81 (101775). 10.1016/j.jenvp.2022.101775.

[R332] Young Deborah Rohm, Hivert Marie-France, Alhassan Sofiya, , 2016. Sedentary behavior and cardiovascular morbidity and mortality: a science advisory from the American heart association. Circulation 134 (13), e262–e279. 10.1161/CIR.0000000000000440. 27528691

[R333] Yu Linling, Liu Wei, Wang Xing, , 2022. A review of practical statistical methods used in epidemiological studies to estimate the health effects of multi-pollutant mixture. Environmental Pollution 306 (August), 119356. 10.1016/j.envpol.2022.119356. 35487468

[R334] Zafeiratou Sofia, Samoli Evangelia, Dimakopoulou Konstantina, , 2021. A systematic review on the association between total and cardiopulmonary mortality/morbidity or cardiovascular risk factors with long-term exposure to increased or decreased ambient temperature. Sci. Total Environ 772, 145383. 10.1016/j.scitotenv.2021.145383. 33578152

[R335] Zafeiratou Sofia, Samoli Evangelia, Analitis Antonis, , 2023. Assessing heat effects on respiratory mortality and location characteristics as modifiers of heat effects at a small area scale in central-northern Europe. Environmental Epidemiology 7 (5), e269. 10.1097/EE9.0000000000000269. 37840857 PMC10569755

[R336] Zafeiratou Sofia, Stafoggia Massimo, Gasparrini Antonio, , 2025. Independent effects of long and short-term exposures to non-optimal increased temperature on mortality. Environmental Pollution 366 (February), 125428. 10.1016/j.envpol.2024.125428. 39617196

[R337] Zhao Qi, Guo Yuming, Ye Tingting, , 2021. Global, regional, and national burden of mortality associated with non-optimal ambient temperatures from 2000 to 2019: a three-stage modelling study. Lancet Planet. Health 5 (7), e415–e425. 10.1016/S2542-5196(21)00081-4. 34245712

[R338] Zhao Tianyu, Heinrich Joachim, Brauer Michael, , 2025. ‘Urban Greenspace under a Changing Climate: benefit or Harm for Allergies and Respiratory Health?’. Environmental Epidemiology 9 (2), e372. 10.1097/EE9.0000000000000372. 39957764 PMC11826049

[R339] Zheng Peng, Afshin Ashkan, Biryukov Stan, , 2022. The burden of proof studies: assessing the evidence of risk. Nat. Med 28 (10), 10. 10.1038/s41591-022-01973-2. PMC9556298 36216935

[R340] Zhou Yan, Yang Liuqi, Yu Jianing, Guo Shiyi, 2022. Do seasons matter? Exploring the dynamic link between blue-green space and mental restoration. Urban For. Urban Green 73 (July), 127612. 10.1016/j.ufug.2022.127612.

[R341] Zijlema Wilma L., Avila-Palencia Ione, Triguero-Mas Margarita, , 2018. Active commuting through natural environments is associated with better mental health: results from the PHENOTYPE project. Environ. Int 121 (December), 721–727. 10.1016/j.envint.2018.10.002. 30321847

